# Antigenic molecular mimicry in viral-mediated protection from cancer: the HIV case

**DOI:** 10.1186/s12967-022-03681-4

**Published:** 2022-10-15

**Authors:** Carmen Manolio, Concetta Ragone, Beatrice Cavalluzzo, Angela Mauriello, Maria Lina Tornesello, Franco M. Buonaguro, Angelo Salomone Megna, Giovanna D’Alessio, Roberta Penta, Maria Tagliamonte, Luigi Buonaguro

**Affiliations:** 1 Innovative Immunological Models Unit, Istituto Nazionale Tumori - IRCCS – “Fond G. Pascale”, Via Mariano Semmola, 52, Naples, Italy; 2 Molecular Biology and Viral Oncogenesis Unit, Istituto Nazionale Tumori - IRCCS – “Fond G. Pascale”, Naples, Italy; 3 Division of Infectious Diseases, AORN San Pio Hospital, Benevento, Italy; 4 Cellular Manipulation and Immunogenetics, Oncology Dep, Ba.S.C.O. Unit, AORN Santobono-Pausilipon, Naples, Italy

**Keywords:** Tumor-associated antigens, Viral antigens, Molecular mimicry, Cross-reactive T cells, HIV-1, Colon cancer, Breast cancer

## Abstract

**Background:**

People living with HIV/AIDS (PLWHA) show a reduced incidence for three cancer types, namely breast, prostate and colon cancers. In the present study, we assessed whether a molecular mimicry between HIV epitopes and tumor associated antigens and, consequently, a T cell cross-reactivity could provide an explanation for such an epidemiological evidence.

**Methods:**

Homology between published TAAs and non-self HIV-derived epitopes have been assessed by BLAST homology. Structural analyses have been performed by bioinformatics tools. Immunological validation of CD8^+^ T cell cross-reactivity has been evaluated ex vivo by tetramer staining.

**Findings:**

Sequence homologies between multiple TAAs and HIV epitopes have been found. High structural similarities between the paired TAAs and HIV epitopes as well as comparable patterns of contact with HLA and TCR α and β chains have been observed. Furthermore, cross-reacting CD8^+^ T cells have been identified.

**Interpretation:**

This is the first study showing a molecular mimicry between HIV antigens an TAAs identified in breast, prostate and colon cancers. Therefore, it is highly reasonable that memory CD8^+^ T cells elicited during the HIV infection may play a key role in controlling development and progression of such cancers in the PLWHA lifetime. This represents the first demonstration ever that a viral infection may induce a natural “preventive” anti-cancer memory T cells, with highly relevant implications beyond the HIV infection.

**Supplementary Information:**

The online version contains supplementary material available at 10.1186/s12967-022-03681-4.

## Introduction

About 40 million people globally were living with HIV in 2020 (PLWHA) and a preventive vaccine is not yet available [1]. All of them have been exposed to multiple HIV epitopes eliciting a specific CD8^+^ T cell immunological memory. Strikingly, while the incidence rates of AIDS-defining cancers are significantly greater in PLWHA than in the general population, non-AIDS-defining cancers show variable standardized incidence ratios (SIRs) compared to the general population [2, 3]. In particular, reduced SIRs for three cancer types, namely breast, prostate and colon cancers have been reported by several meta-analyses [4–7]. So far, no biological explanations have been provided for such epidemiological evidence. A reduced implementation of population screenings has been proposed. But this seems to be unlikely, given that the lower risk is present for both early-stage tumors, primarily screen-detected, and larger tumors, clinically detected.

In the present study we wanted to verify whether the molecular mimicry between HIV antigens and TAAs expressed by breast, prostate and colon cancers could explain the reduced SIRs in PLWHA. In a word, whether the HIV infection could represent a natural “anti-cancer vaccination”.

Indeed, viruses are a natural source of non-self antigens (VirAs) expressed by host’s cells in the context of the HLA class I molecules, inducing a wide pool of specific memory CD8^+^ T cell clones. We and others have previously shown that the molecular mimicry between viral and tumor antigens may have a significant impact in controlling tumor growth and improving the clinical outcome in cancer patients [8–11]. More recently, we have shown that TAAs may show sequence homology as well as structural similarities with viral peptides and cross-reacting CD8 + T cells can be identified to drive the fate of cancer development and progression [12]. Therefore, a previous viral infection may represent a natural “anti-cancer vaccination” and anti-viral memory CD8 + T cells may be promptly recalled by cancer cells expressing TAAs similar or identical to VirAs. This is possible because the degeneracy of the TCR in antigen recognition allows each single receptor to cross-react against similar antigens, recognizing at least 10^6^ different MHC-bound peptides [13, 14].

Indeed, striking sequence homologies between multiple TAAs and HIV epitopes have been found. High structural similarities between the paired TAAs and HIV epitopes as well as comparable patterns of contact with HLA and TCR α and β chains have been observed. Furthermore, cross-reacting CD8^+^ T cells have been identified with shared sequences in TCR α and β chains.

Overall, it is highly reasonable that memory CD8 + T cells elicited by HIV epitopes may play a key role in controlling development and progression of such cancers in the PLWHA lifetime.

## Materials and methods

### Protein expression analysis

Starting from downloadable data section of Human Protein Atlas database (https://www.proteinatlas.org/about/download), Normal Tissue and Pathology dataset were downloaded. They contain expression profiles evaluated by immunohistochemistry on tissue micro array of proteins of different human tissues samples, non tumoral and tumoral respectively.

Normal Tissue dataset is an extremely wide list containing information about the expression of several proteins in different cell types of a specific human tissue. To identify tumor specific proteins, only those proteins who were defined as “not detected” in normal cells from the same tissues were selected. Subsequently, these proteins were searched in the Pathology dataset to select only those detected in “colon, breast, prostate, lung or brain cancer (ca)” tissue samples at “high”, “medium” or “low” expression level. The definition of expression levels was according to Human Protein Atlas database.

### HLA class l epitope prediction

In order to predict MHC-class I epitopes, protein sequences were downloaded from UniProt database (https://www.uniprot.org/) and the entire sequence was analyzed with NetMHCpan4.1 tool (https://services.healthtech.dtu.dk/service.php?NetMHCpan-4.1), to predict the binding affinity [15]. Prediction analyses were performed for the 12 most frequent alleles in the world, selecting only peptides considered weak and strong binders, according to default parameters (WB; SB).

### BLAST homology search

Peptides selected as SB according to NetMHCpan 4.1 prediction tool have been submitted to BLAST for a homology search against HIV-1 (taxid: 11,676) within the non-redundant protein sequences database (https://blast.ncbi.nlm.nih.gov/Blast.cgi). HIV-derived homologous sequences were analyzed to predict their binding affinity and stability to all alleles as described above.

### Epitope modelling and molecular docking

The 3D structure of interaction between peptides and HLA alleles was generated using Pymol software (PyMol Molecular graphics system, version 1.8.6.2) and Molsoft ICM (http://www.molsoft.com/, version 3.8-7d) software. The PDB format of complex between HLA molecules and reference peptides were downloaded from RCS Protein Data Bank (PDB) website (https://www.rcsb.org). In particular, HLA-A*01:01 (3BO8), HLA-A*02:01 (1AO7), HLA-A*03:01 (3RL1), HLA-A*24:02 (7JYV), HLA-B*07:02 (7LGD), HLA-B*08:01 (7NUI), HLA-B*15:01 (6VB3), HLA-B*39:01 (4O2E), HLA-B*40:02 (5IEK), HLA-B*58:01 (5VWH). The original peptide sequences in the downloaded structures were replaced with individual predicted epitopes using Pymol visualization system and Molsoft ICM software was used to visualize the molecular docking.

### Peptide synthesis and solubilization

All peptides were synthesized at a purity of ≥ 90% (GenScript; NJ, USA). Lyophilized powders were reconstituted in DMSO Solution (CARLO ERBA Reagents S.r.l., Cornaredo; Italy) and diluted in 90% of 1X PBS (HyClone, Thermo Fisher Scientific Inc., US).

### Peptide binding affinity and BFA decay assays

Peptide binding affinity to HLA-A*02:01 molecule and BFA decay assays were performed for each candidate peptide. Human TAP-deficient T2 cell line (174xCEM.T2; ATCC CRL 1992™) was purchased from American Type Culture Collection (ATCC; https://www.atcc.org/) and cultured in Iscove's modified Dulbecco's medium (IMDM; Gibco Life Technologies) containing 25 mM HEPES and 2 mM L-Glut, supplemented with 20% fetal bovine serum (FBS; Capricorn Scientific GmbH), 100 IU/ml penicillin and 100 μg/ml streptomycin (Gibco Life Technologies). Cells were maintained at 37 °C in a humidified incubator with 5% CO_2_. Briefly, T2 were seeded at 3.5 × 10^5^ cells per well in 24 well plates and incubated 16 h at 27°C with peptides (final concentrations: 5, 10, 20, 50 and 100 μM) in IMDM serum free medium. The next day, cells have been incubated for additional 2 h at 37 °C. Following incubation, cells were harvested and centrifuged at 200 × g for 5 min. Subsequently, cells were washed twice with phosphate buffered saline (1X PBS; Gibco Life Technologies) and stained with R-PE conjugated anti human HLA-A2 mono-clonal antibody (cat. 343,306; BioLegend), for 30 min at 4 °C, and analyzed with the Attune™ NxT flow cytometer (Thermo Fisher Scientific). Mouse H-2 Kb-specific OVA SIINFEKL peptide was used as negative control and T2 cells without any added peptide were used as a background control. A fluorescence index (FI) was calculated using the following formula: FI = [mean fluorescence intensity (MFI) sample – MFI background]/MFI background, where MFI background represents the value without peptide. A value of FI > 0.5 was set as threshold to indicate peptides with affinity for the HLA-A*02:01 molecule. For the brefeldin-A decay assay, T2 were incubated with peptides (50 μM) as described above, washed and treated with 1X BFA (brefeldin A solution; cat. 420,601; BioLegend) in IMDM serum free medium, for 1 h at 37°C. Cells were harvested every two hours (T0, T2, T4, T6, T8), washed with phosphate buffered saline (1X PBS; Gibco Life Technologies), stained with anti HLA-A*0201 fluorescent monoclonal antibody (cat. 343,306; BioLegend) and analyzed by flow cytometry. The stability of each peptide bound to HLA-A*02:01 was measured as the DC_50_ value, which was defined as an estimate of the time required for a 50% reduction of the MFI value at time 0. The DC_50_ value was calculated according to the formula: MFI at indicated time points/MFI at time 0 × 100. All the experiments were performed in triplicate [11, 16].

### pMHC multimer preparation and T cell staining

pMHC complexes were generated by combining purified disulfide-stabilized HLA-A*0201 monomer (100 μg/ml) with 100 μM peptide for 30 min. in PBS at room temperature [17]. pMHC complexes were centrifuged for 5 min. at 3300 × g to sediment any aggregated MHC molecules. For each 100 μl pMHC, 9.02 μl (0.2 mg/ml stock, SA-PE (Bio-legend, 405,204), SA-APC (Biolegend, 405,207), SA-PE/Cy7 (Biolegend, 405,206), SA-PE-CF594 (BD, 562,284)) or 18.04 μl (0.1 mg/ml stock, SA-BV421 (Biolegend, 405,226), SA-BV650 (Biolegend, 405,231) streptavidin-conjugate was added and incubated for 30 min. on ice. D-biotin (Sigma-Aldrich) was added at a final concentration of 25 μM to block any free binding sites and multimers were stored at − 20 °C with 5% glycerol and 0.5% BSA. To stain for T cell reactivity, PBMCs from 16 HIV-1 patients and 10 healthy donors (2–5 × 10^6^) were incubated with a pool of pMHC multimers (3 μl/multimer) and dasatinib (50 nM final, LC laboratories, D-3307) for 15 min. at 37 °C. Cells were then stained with antibodies CD3-FITC (1:40, BD, 345,763) and CD8-BV480 (1:100, BD, 566,121) and LIVE/DEAD Fixable Near-IR (1:1000, Invitrogen, L10119) for 30 min. on ice and washed twice in FACS buffer (PBS + 2% FCS). Gating for CD3^+^/CD8^+^ T cells was done on alive cells and binding to pMHCs was assessed by measuring specific fluorescence associated with each individual pMHC. Samples were acquired on a flow cytometer (LSRFortessa, BD) and analyzed by the Attune NxT software v3.1.2 (ThermoFisher scientific).

### Statistical analysis

Comparison between individual data points were performed with the two-sided Student’s t-test and ANOVA, as appropriate. Paired, for the matched samples collected at the Istituto Nazionale Tumori—IRCCS—"Fond G. Pascale", Naples, Italy (INT); unpaired for the samples extracted by the publicly available data (http://gent2.appex.kr/gent2/). Normally distributed data were represented as mean ± S.E.M. Two-way ANOVA and Bonferroni post-hoc analysis were used to examine the significance of differences among groups. All P values were two-tailed and considered significant if less than 0.05.

## Results

### Identification of tumor-related proteins

In order to identify proteins specifically overexpressed in breast, prostate and colon cancers and not detected in normal cells, the Normal Tissue dataset (15,170 unique proteins) and the Pathology dataset (15,313 unique proteins) available at the Human Protein Atlas were sequentially interrogated (https://www.proteinatlas.org/about/download). The same analysis was performed for the lung and brain cancers, which show increased SIRs in PLWHA [5].

An average of 5,768 proteins showed no expression in normal cells of the five tissues, with a range from 4,814 (breast glandular cells) to 7,019 (lung pneumocytes). In parallel, an average of 4,134 proteins were found to be detected in cancer cells, with a range from 2,640 (lung ca) to 5,650 (colon ca). Of the latter proteins, an average of 213 proteins (5.15%) were found to be at high levels in cancer cells, as defined by the Human Protein Atlas, with a range from 86 (lung ca) to 408 (colon ca). Cancer-specific proteins were selected for each tissue as those not detected in normal cells and highly expressed in cancer cells,. The number of the latter proteins ranged from 1 to 2 for breast, prostate, lung and brain tumors. The only exception was the colon cancer for which 44 cancer-specific proteins were identified (Table 1). The subsequent filter was set for selecting proteins not detected in > 70% of normal cells of any tissue. Indeed, only such proteins should not be affected by immune tolerance impairing the immune system to mount responses against self antigens. According to such additional filter, six cancer-specific proteins in colon cancer (CEACAM8, CDH17, GLOD5, PPM1E, TRIM16, EPCAM) and one in breast cancer (CCR9) were identified. None of the cancer-specific proteins identified in prostate cancer, brain and lung cancer met these parameters (Additional file 1: Fig. S1). Nevertheless, they were selected for the subsequent analyses.

**Table 1:** Number of proteins identified in normal and cancer cells of indicated tissues. Cancer specific proteins are defined in the text

Tumor	Normal	Cancer		Cancer specific
	N.D	All	High	
Breast	4814	4997	219	1
Colon	5823	5650	408	44
Prostate	5036	4543	187	1
Lung	7019	2640	86	2
Brain	6151	2841	169	2
Average	5768.6	4134.2	213.8	

### Prediction of Tumor-associated antigens—TAAs

The cancer-specific proteins selected in each cancer were submitted to the NetMHCpan software to predict 9 aa-long epitopes (nonamers) strong binders (SBs) to HLA alleles covering more than 70% of the World population. The analysis was performed considering all the overlapping nonamers with a one-amino-acid lateral shift covering the entire protein sequence. In order to select the best predicted epitopes, we focused our analysis only on those showing an affinity < 100 nM to HLA molecules, which we have been previously shown to have a 100% concordance with ex vivo binding assay [11, 12, 16].

**Fig. 1:**
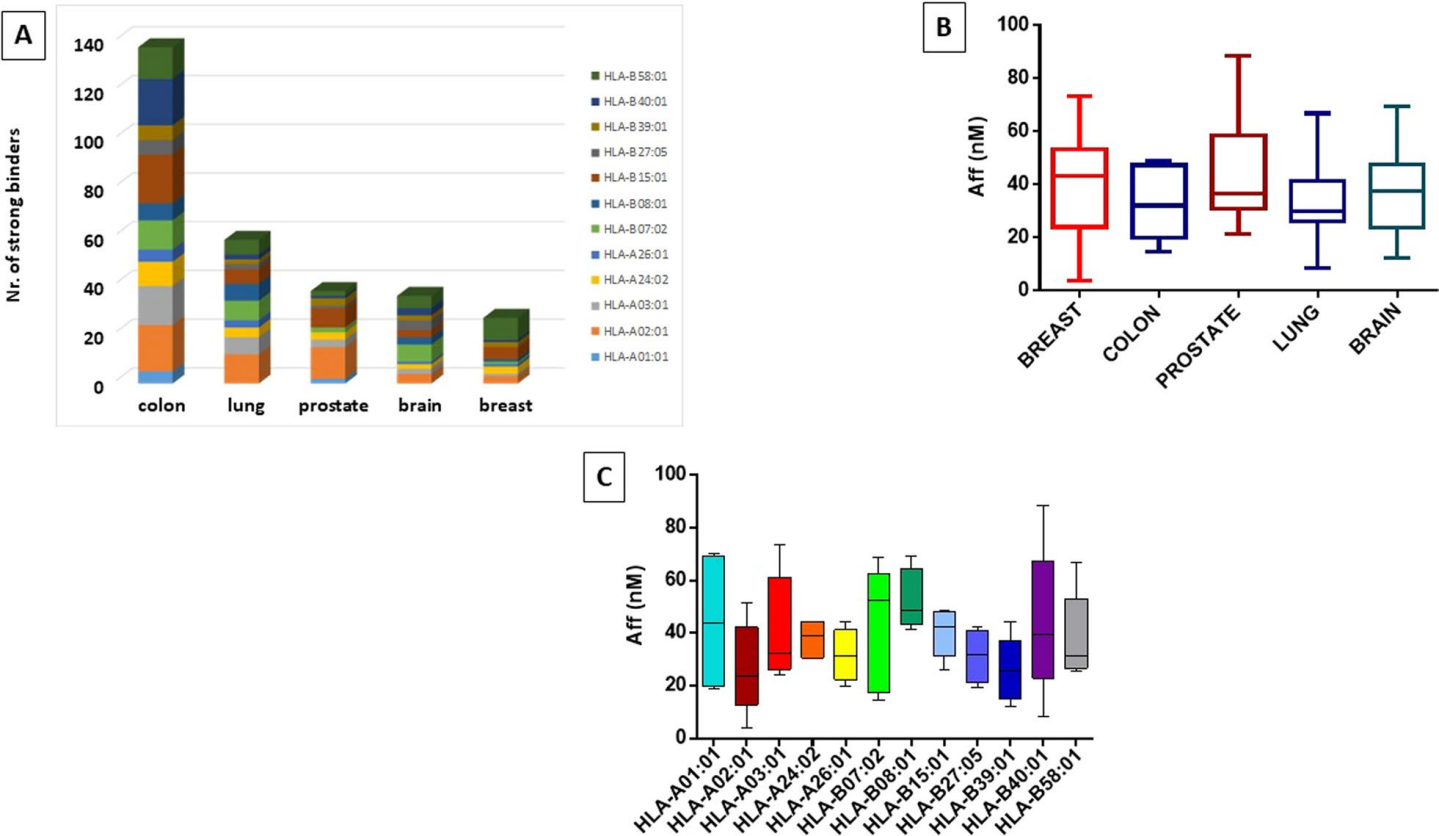
Predicted tumor antigens. Cumulative number of predicted antigens from all cancer-specific proteins identified in each tumor, listed by cancer (**A**). Cumulative predicted affinity of predicted antigens from all cancer-specific proteins identified in each tumor, listed by cancer (**B**) and by HLA allele (**C**)

The number of SBs with such characteristics predicted from the cancer-specific proteins was highly variable among the five cancers. The highest number of SBs (138) was predicted in colon cancers from the 6 cancer-specific proteins, covering all the evaluated HLA alleles; the lowest number [27] was predicted in breast cancer from the single CCR9 cancer-specific protein (Fig. 1A). Regardless the different numbers of SBs predicted for the cancer-specific proteins from the 5 cancers, their average affinity to the HLA alleles was very high (e.g. < 50 nM) (Fig. 1B). The number of SBs specific to each HLA allele was highly variable. The highest number was predicted for HLA-A*02:01 (51), the second and third highest numbers were predicted for HLA-B*15:01 (42) and HLA-B*58:01 (35). On the contrary, the lowest numbers by far were predicted for HLA-A*01:01 [7] and HLA-A*26:01 [10]. The number of predicted SBs for all other HLA alleles ranged between 13 and 30 (Additional file 1: Fig. S2). Furthermore, the average affinity to the HLA alleles was highly variable without reaching a statistical difference. The highest average affinity was observed for HLA-A*02:01 specific epitopes (e.g. 26 nM), the lowest was observed for HLA-B*08:01 specific epitopes (e.g. 52 nM) (Fig. 1C).

In order to verify the expression of the predicted SBs on normal cells, the HLA Ligand Atlas was interrogated. Interestingly, 271 (93.7%) of the 298 SBs predicted in the cancer-specific proteins were found not expressed in the HLA ligandome of normal cells (Table 2). All peptides found at the HLA Ligand Atlas were excluded from subsequent analyses. Moreover, some of such predicted TAAs have been previously described to be presented by cancer cells and/or be target of T cells [18–22].

**Table 2:** Predicted TuAs from **cancer-specific proteins** and their presence in the HLA Ligand Atlas of normal tissues

MHC	Peptide	Aff (nM)	HLA Ligand Atlas
*Breast: CCR9*			
HLA-A*02:01	AIADLLFLV	3,76	No match
HLA-A*02:01	FLPPLYWLV	2,67	No match
HLA-A*02:01	ILGFFLPFV	5,12	No match
HLA-A*03:01	KSSKHKALK	73,36	No match
HLA-A*24:02	LYVFVGERF	22,26	No match
HLA-A*24:02	CYTIIIHTL	40,22	No match
HLA-A*24:02	RYIAIAQAM	70,57	No match
HLA-A*26:01	QTIDAYAMF	19,7	No match
HLA-B*07:02	TPTDFTSPI	68,5	No match
HLA-B*08:01	STKLKSAVL	48,06	No match
HLA-B*39:01	FHSCLNPVL	5,92	No match
HLA-B*39:01	SQFPYNCIL	45,12	No match
HLA-B*40:01	MEDYVNFNF	46,12	No match
HLA-B*58:01	IAAADQWKF	13,32	No match
HLA-B*58:01	NSLVILVYW	28,08	No match
HLA-B*58:01	VTITVLTVF	48,71	No match
HLA-B*58:01	LTLKVILGF	31,09	No match
HLA-B*58:01	WAIAAADQW	7,46	No match
HLA-B*58:01	LGCISQAQW	22,22	No match
HLA-B*58:01	KTMTDMFLL	10,03	No match
HLA-B*58:01	SSMEDYVNF	96,02	No match
HLA-B*58:01	LTVFVLSQF	92,21	No match
HLA-B*15:01	FQVTQTIAF	11,07	No match
HLA-B*15:01	LLVQTIDAY	23,98	No match
HLA-B*15:01	LLYSKMVCF	42,55	No match
HLA-B*15:01	RVKTMTDMF	94,33	No match
HLA-B*15:01	VTITVLTVF	39,98	No match
*Brain: DPYSL2*			
HLA-A*02:01	KMDENQFVA	37,21	Ovary
HLA-A*02:01	VLEDGTLHV	56,91	No match
HLA-A*02:01	ALAGGTTMI	98,73	No match
HLA-A*03:01	KVFNLYPRK	11,63	No match
HLA-A*03:01	ITSDRLLIK	83,95	No match
HLA-A*24:02	VYMAFKDRF	8,25	Liver, Lung
HLA-A*26:01	HVTEGSGRY	64,02	No match
HLA-B*07:02	YPRKGRIAV	2,87	Liver, Lung
HLA-B*07:02	IPRRTTQRI	19,79	No match
HLA-B*07:02	IPRITSDRL	26,75	No match
HLA-B*07:02	IPRKPFPDF	27,37	No match
HLA-B*07:02	KARSRLAEL	37,6	No match
HLA-B*07:02	SAKTHNSSL	74,97	No match
HLA-B*07:02	VPEPGTSLL	88,37	No match
HLA-B*08:01	SAKTHNSSL	40,66	No match
HLA-B*08:01	YPRKGRIAV	54,2	No match
HLA-B*15:01	FQLTDCQIY	53,36	No match
HLA-B*15:01	KTHNSSLEY	78,35	Tongue
HLA-B*27:05	KRIKARSRL	70,69	No match
HLA-B*27:05	ARSRLAELR	76,74	No match
HLA-B*27:05	SRMVIPGGI	76,85	No match
HLA-B*40:01	LEYNIFEGM	42,86	Multiple Tissues
HLA-B*40:01	IEAHSRMVI	86,33	No match
HLA-B*40:01	MEDGLIKQI	95,51	Multiple Tissues
HLA-B*58:01	AAFDQWREW	26,57	No match
HLA-B*58:01	LGTDGSHYW	27,85	No match
HLA-B*58:01	GSDADLVIW	38,13	No match
HLA-B*58:01	SLLAAFDQW	53,69	No match
*Brain: SPARC*			
HLA-A*02:01	WLKNVLVTL	38,11	No match
HLA-A*24:02	YIFPVHWQF	79,77	Multiple tissues
HLA-B*08:01	WLKNVLVTL	91,35	No match
HLA-B*15:01	KTFDSSCHF	29,21	No match
HLA-B*27:05	MRAWIFFLL	9,57	No match
HLA-B*39:01	MRDWLKNVL	8,7	No match
HLA-B*39:01	MRAWIFFLL	15,81	No match
HLA-B*58:01	KTFDSSCHF	14,61	No match
*Colon: CDH17BIS*			
HLA-A*01:01	VIEREGLLY	70,39	No match
HLA-A*01:01	FTGSSKILY	43,81	No match
HLA-A*01:01	NTANSFLNY	46,99	No match
HLA-A*02:01	ILQAHLHSL	12,25	No match
HLA-A*02:01	KLPRFPFSI	14,74	No match
HLA-A*02:01	IQLPMINNV	54,97	No match
HLA-A*02:01	LVIGIILAV	32,48	No match
HLA-A*02:01	GLFLIQTYA	43,93	No match
HLA-A*03:01	VMYFQINNK	10,12	No match
HLA-A*03:01	QLAKQSLKK	72,8	No match
HLA-A*03:01	AVVFIRIKK	79,68	No match
HLA-A*24:02	LFRGPHFTF	71,32	No match
HLA-A*26:01	NTANSFLNY	56,93	No match
HLA-B*07:02	HPLSAPGSL	11,9	No match
HLA-B*07:02	RPAGHQTGI	13,94	No match
HLA-B*07:02	APQFSQHVF	55,95	No match
HLA-B*07:02	RPGKPFLYV	59,59	No match
HLA-B*08:01	ILQAHLHSL	37,38	No match
HLA-B*08:01	MLQLAKQSL	92,53	No match
HLA-B*39:01	THNLQVAAL	16,43	No match
HLA-B*39:01	FKAENPEPL	13,19	No match
HLA-B*40:01	RETRSTHNL	35,21	No match
HLA-B*40:01	FEVQENERL	42,52	No match
HLA-B*40:01	GETDNIFVI	12,73	No match
HLA-B*40:01	FEEREYVVL	18,94	No match
HLA-B*40:01	GEIFSVAPL	5,02	No match
HLA-B*40:01	FETAAVSNI	52,35	No match
HLA-B*40:01	QEGKFSGPL	41,12	No match
HLA-B*58:01	KANPPAVTF	17,57	No match
HLA-B*58:01	LSAPGSLIF	12,51	No match
HLA-B*58:01	GSGSLQNDW	83,99	No match
HLA-B*58:01	SSLSSVSEF	88,68	No match
HLA-B*15:01	RLSTRHTEF	44,77	No match
HLA-B*15:01	GVKYNASSF	67,45	No match
HLA-B*15:01	LSAPGSLIF	34,98	No match
*Colon: GLOD 5* *MHC*			
HLA-A*02:01	RLDHIVMTV	5,77	No match
HLA-A*02:01	NLIEVSNYI	10,19	No match
HLA-A*03:01	KMWGRTLEK	3,88	No match
HLA-A*03:01	IVMTVKSIK	55,26	No match
HLA-B*08:01	MLRHLPSRL	90,51	No match
HLA-B*58:01	KSIKDTTMF	9,83	No match
HLA-B*15:01	SIKDTTMFY	60,19	No match
*Colon: PPM1E*			
HLA-A*01:01	CSAPADLGY	31,65	No match
HLA-A*02:01	SVFSKLHEI	36,35	No match
HLA-A*02:01	FLAAALARA	5,94	No match
HLA-A*02:01	YLDLTQIEA	57,91	No match
HLA-A*02:01	SLSPVCSGL	61,33	No match
HLA-A*03:01	KLARSVFSK	9,37	No match
HLA-A*03:01	AIYASIHLH	98,98	No match
HLA-A*03:01	LINELMMEK	59,44	No match
HLA-A*03:01	VVFLRDMNK	32,23	No match
HLA-A*03:01	HLRHHYSKK	46,87	No match
HLA-A*03:01	RIRSSLPWR	53,88	No match
HLA-A*24:02	RFNPKFYSF	11,44	No match
HLA-A*24:02	HYSKKWHRF	20	No match
HLA-A*24:02	TYRRFLELF	14,81	No match
HLA-A*24:02	IYASIHLHV	38,63	No match
HLA-A*24:02	YYETSIHAI	83,38	No match
HLA-A*24:02	LYKYNCPSF	35,21	No match
HLA-B*07:02	FPLRRRPQL	9,87	No match
HLA-B*07:02	FPHDPAEAL	63,53	No match
HLA-B*07:02	SPGNRVSRL	35,08	No match
HLA-B*07:02	SPGSQINVL	51,81	No match
HLA-B*07:02	KPHSAQFLL	47,07	No match
HLA-B*07:02	LPRPLSERI	66,63	No match
HLA-B*07:02	CPSFLAAAL	22,27	No match
HLA-B*08:01	FPLRRRPQL	9,91	No match
HLA-B*08:01	MLVRKGQAV	26,5	No match
HLA-B*08:01	KTYRRFLEL	92,81	No match
HLA-B*15:01	LQSDLSAHY	11,02	No match
HLA-B*15:01	ASKPHSAQF	74,89	No match
HLA-B*15:01	AQFLLPVEM	90,51	No match
HLA-B*15:01	RLSHLRHHY	67,82	No match
HLA-B*27:05	HRFRFNPKF	21,58	No match
HLA-B*27:05	KRNRIRSSL	47,8	No match
HLA-B*27:05	YRRFLELFL	52,81	No match
HLA-B*39:01	WRVNGSLSV	42,89	No match
HLA-B*39:01	YRMQSLSPV	16,71	No match
HLA-B*40:01	GEFPTAFNL	8,41	No match
HLA-B*40:01	REEVEGESL	22,28	No match
HLA-B*40:01	LEDPGYLDL	59,85	No match
HLA-B*40:01	QEEQAYFAV	54,53	No match
HLA-B*58:01	SVQSSLPEW	44,83	No match
HLA-B*58:01	RGNMLHVAW	34,54	No match
*Colon: TRIM16*			
HLA-A*02:01	YLHRYYFEV	2,33	No match
HLA-A*02:01	VLSQQSLYL	58,02	No match
HLA-A*02:01	RMAAISNTV	13,5	No match
HLA-A*03:01	GTYVGLTCK	29,93	No match
HLA-A*03:01	SVYVGLKDK	83,52	No match
HLA-A*24:02	RYYFEVEIF	10,7	No match
HLA-A*26:01	DTMTLVHKF	63,32	No match
HLA-A*26:01	NTVQFLEEY	32,67	No match
HLA-B*08:01	SLYLHRYYF	95,86	No match
HLA-B*15:01	LQYAYDITF	8,56	No match
HLA-B*15:01	HLIQLLENY	64,75	No match
HLA-B*15:01	RQVLSQQSL	84,65	No match
HLA-B*15:01	ILSFYGVEY	57,78	No match
HLA-B*15:01	SLYLHRYYF	75,83	No match
HLA-B*27:05	RRLGVYIDF	56,62	No match
HLA-B*27:05	SRFLHWRQV	54,5	No match
HLA-B*40:01	AEMQFGELL	4,59	Thymus
HLA-B*40:01	SEVKAVAEM	28,88	No match
HLA-B*40:01	LEEKEQAAL	40,08	No match
HLA-B*40:01	QEHSGHTIV	61,14	No match
HLA-B*40:01	QELERMAAI	97,6	No match
HLA-B*58:01	KAQANVMLF	13,36	No match
HLA-B*58:01	CISGNNFSW	31,21	No match
*Colon: CEACAM8*			
HLA-A*01:01	FSDPVTLNV	44,46	Bone marrow
HLA-A*02:01	IMIGVLARV	5,67	No match
HLA-A*02:01	FSDPVTLNV	65,43	No match
HLA-A*02:01	KLNLMSEEV	39,39	No match
HLA-A*03:01	GTFQQYTQK	35,5	Bone marrow
HLA-A*03:01	GSYTLQVIK	70,59	No match
HLA-A*24:02	LYGPDAPTI	83,9	No match
HLA-A*24:02	IYPNASLLM	53,31	No match
HLA-A*26:01	ETIYPNASL	71,49	No match
HLA-B*07:02	LPVSPRLQL	9,45	No match
HLA-B*15:01	QQITPGPAY	19,97	Bone marrow
HLA-B*15:01	IQNPASANF	9,34	Bone marrow
HLA-B*15:01	SVNGTFQQY	83,79	Bone marrow
HLA-B*15:01	FQQYTQKLF	52,48	No match
HLA-B*15:01	VTRNDTGSY	94,41	No match
HLA-B*27:05	WRIPWQGLL	18,87	No match
HLA-B*39:01	YHAGVNLNL	9,24	No match
HLA-B*39:01	WRIPWQGLL	91,33	No match
HLA-B*40:01	AEGKEVLLL	78,97	No match
HLA-B*58:01	LTASLFTFW	3,13	No match
HLA-B*58:01	ETQNTTYLW	36,36	No match
HLA-B*58:01	YSWSVNGTF	20,84	No match
*Colon: EPCAM*			
HLA-A*02:01	GLKAGVIAV	69,41	No match
HLA-A*02:01	VVAGIVVLV	48,75	No match
HLA-A*03:01	KSLRTALQK	50,33	No match
HLA-A*26:01	DIADVAYYF	90,16	No match
HLA-B*40:01	KEITTRYQL	25,58	No match
HLA-B*40:01	AEIKEMGEM	66,18	No match
HLA-B*58:01	CSERVRTYW	23,26	No match
HLA-B*15:01	LQKEITTRY	14,52	No match
HLA-B*15:01	LLAAATATF	4,39	No match
*Prostate: NDUFS2*			
HLA-A*01:01	VTAEEALNY	58,86	No match
HLA-A*01:01	VSDGSSRPY	72,62	No match
HLA-A*02:01	RLLNHIMAV	2,59	No match
HLA-A*02:01	KMFEFYERV	3,05	No match
HLA-A*02:01	KLIEYKTYL	4,96	No match
HLA-A*02:01	MLADVVAII	5,94	No match
HLA-A*02:01	KLYTEGYQV	6,73	No match
HLA-A*02:01	YQFSKNFSL	7,18	Multiple tissues
HLA-A*02:01	HMLADVVAI	16,16	No match
HLA-A*02:01	VLFGEITRL	19,13	No match
HLA-A*02:01	ALNYGFSGV	23,23	No match
HLA-A*02:01	IMAVTTHAL	27,34	No match
HLA-A*02:01	RLDELEELL	29,47	No match
HLA-A*02:01	GLMDDIYQF	49,65	No match
HLA-A*02:01	KMPPGEIKV	84,76	No match
HLA-A*03:01	KTYLQALPY	18,63	No match
HLA-A*03:01	RIIAQCLNK	25,84	No match
HLA-A*03:01	GLLHRGTEK	52,91	No match
HLA-A*24:02	TYLQALPYF	10,57	No match
HLA-A*24:02	AMTPFFWLF	20,73	No match
HLA-A*24:02	RYLCRVEEM	59,38	No match
HLA-B*07:02	RPGGVHQDL	21,45	No match
HLA-B*07:02	RVEEMRQSL	90,96	No match
HLA-B*15:01	YQVPPGATY	9,56	No match
HLA-B*15:01	AQQFGGAVM	15,81	No match
HLA-B*15:01	SMMCNEQAY	16,28	No match
HLA-B*15:01	QQFGGAVMY	23,49	No match
HLA-B*15:01	KTYLQALPY	41,5	No match
HLA-B*15:01	YQFSKNFSL	56,91	No match
HLA-B*15:01	IVKNITLNF	58,22	No match
HLA-B*15:01	SLIHHFKLY	68,72	No match
HLA-B*27:05	ARMHAAYIR	37,13	No match
HLA-B*39:01	YQFSKNFSL	5,33	No match
HLA-B*39:01	FRGVAAQVL	45,59	No match
HLA-B*39:01	THALDLGAM	81,98	No match
HLA-B*40:01	AEEALNYGF	88,35	No match
HLA-B*58:01	LGAMTPFFW	6,09	No match
HLA-B*58:01	RAQWIRVLF	56,87	No match
*Lung: CNIH4*			
HLA-A*02:01	LLMSLHWFI	3,25	No match
HLA-A*02:01	YLYSMILAL	3,82	No match
HLA-A*02:01	SLHWFIFLL	10,12	No match
HLA-A*02:01	FLLNLPVAT	15,76	No match
HLA-A*02:01	AMIKLGFHL	21,24	No match
HLA-A*02:01	LIGHTIVTV	69,56	No match
HLA-A*24:02	LYSMILALI	32,32	No match
HLA-B*07:02	VPSGNMGVF	77,38	No match
HLA-B*08:01	MIKLGFHLL	25,27	No match
HLA-B*58:01	LLNLPVATW	99,66	No match
*Lung: PRPF40A*			
HLA-A*02:01	YLMDNPTFA	4,03	No match
HLA-A*02:01	ALDLFKFYV	8,07	No match
HLA-A*02:01	MMMSHMSQA	9,12	No match
HLA-A*02:01	RIFKDFMHV	15,96	No match
HLA-A*02:01	NILDNMANV	34,74	No match
HLA-A*02:01	TLDAGNIKL	84,81	Ovary
HLA-A*03:01	KMTSTTRYK	11,36	No match
HLA-A*03:01	KSNLHAMIK	29,28	No match
HLA-A*03:01	IVAGSLITK	36,6	Multiple tissues
HLA-A*03:01	KQAFKELLK	63,33	No match
HLA-A*03:01	LAFNSLLEK	65,36	No match
HLA-A*03:01	TVADFTPKK	91,15	No match
HLA-A*03:01	MTSTTRYKK	98,8	No match
HLA-A*24:02	SWMELYPTI	12,79	No match
HLA-A*24:02	QYLMDNPTF	54,19	No match
HLA-A*24:02	RYKKAEQMF	69,86	No match
HLA-A*26:01	EIPTTMSTM	42,38	No match
HLA-A*26:01	EIYEDVLFF	48,08	No match
HLA-A*26:01	FVVEVNTTF	90,52	Multiple tissues
HLA-B*07:02	HPMGQRANM	5,68	No match
HLA-B*07:02	RPSMGHPGM	6,99	No match
HLA-B*07:02	HPGMHYAPM	8,62	No match
HLA-B*07:02	MPGMMSSVM	11,35	No match
HLA-B*07:02	MPPMGGPPM	28,69	Spleen
HLA-B*07:02	MPPVPHGMM	60,4	No match
HLA-B*07:02	VPHGMMPQM	70,06	No match
HLA-B*08:01	NIKLAFNSL	30,71	No match
HLA-B*08:01	ELEKRRRTL	57,75	No match
HLA-B*15:01	RQRKNRESF	38,7	No match
HLA-B*15:01	FVVEVNTTF	39,02	No match
HLA-B*15:01	KMKRKESAF	42,12	No match
HLA-B*15:01	SMGHPGMHY	45,42	No match
HLA-B*15:01	SMSSWMELY	55,07	No match
HLA-B*15:01	SQASMQPAL	93,92	No match
HLA-B*27:05	RRRTLLEQL	35,63	No match
HLA-B*27:05	SRWAKPKEL	70,18	No match
HLA-B*39:01	SQASMQPAL	33,18	No match
HLA-B*39:01	NRESFQIFL	87,31	No match
HLA-B*40:01	GEMEVWNAI	5,84	No match
HLA-B*40:01	KESAFKSML	27,62	No match
HLA-B*58:01	GSSLCSGSW	22,83	No match
HLA-B*58:01	QMFGEMEVW	23,31	No match
HLA-B*58:01	KTGKDSGNW	25,86	No match
HLA-B*58:01	TASGAKSMW	39,94	No match
HLA-B*58:01	STALDLFKF	58,82	No match

### Identification of homologous HIV-1 epitopes

**Fig. 2:**
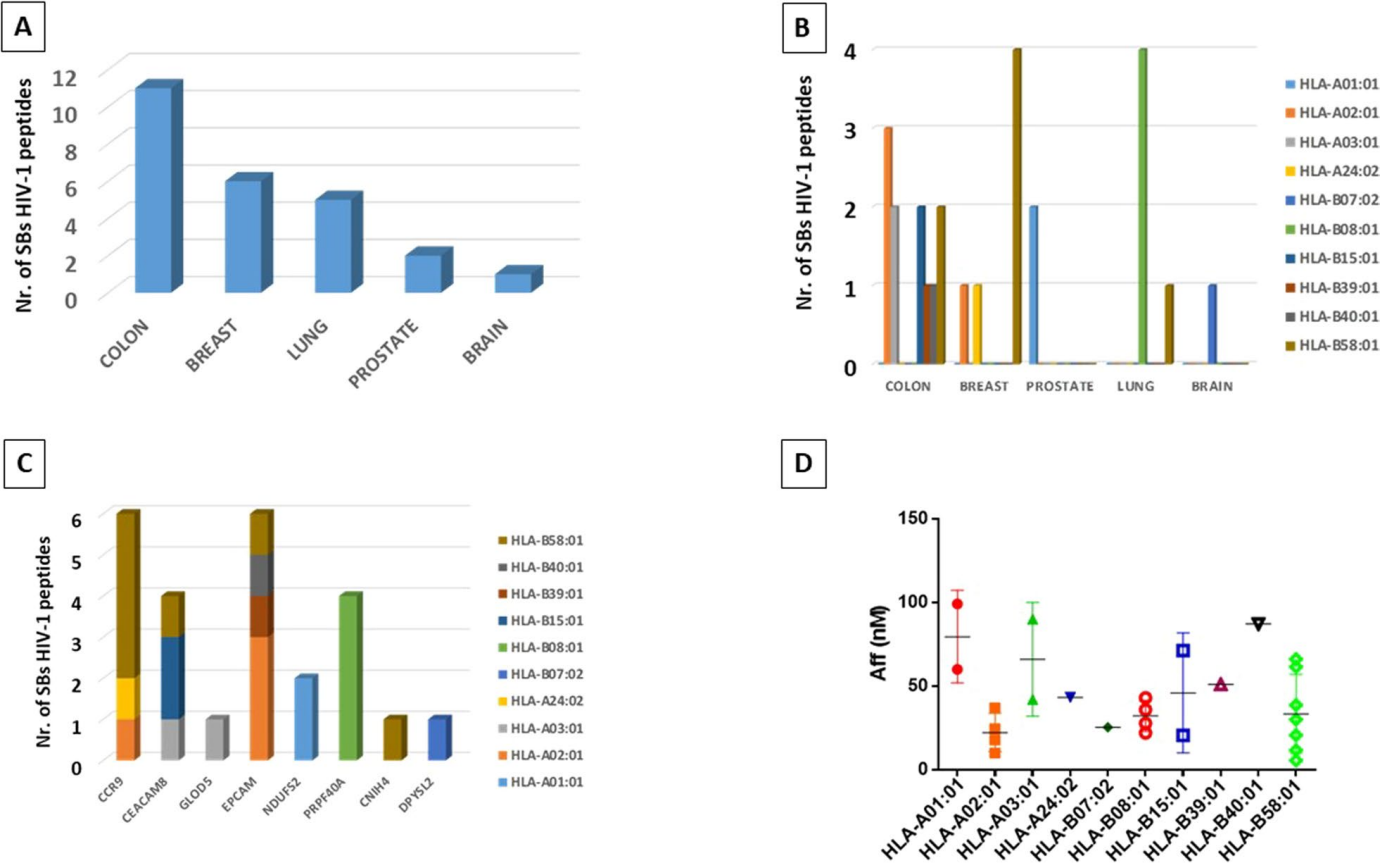
Predicted HIV-1 peptides homologous to tumor antigens. Cumulative number of HIV-1 predicted antigens homologous to TAAs from all cancer-specific proteins identified in each tumor (**A**). Number of HIV-1 predicted antigens associated to each HLA allele homologous to TAAs from all cancer-specific proteins identified in each tumor, listed by cancer (**B**). Number of HIV-1 predicted antigens associated to each HLA allele homologous to TAAs from each cancer-specific proteins identified in each tumor, listed by protein (**C**). Cumulative predicted affinity of HIV-1 predicted antigens listed by HLA allele (**D**)

The following step was to verify the molecular mimicry between TAAs predicted for the cancer-specific proteins and HIV-1 antigens. Indeed, this might explain a cross-protective anti-cancer immune response elicited by HIV-1 antigens in HIV-1 patients. To this aim, the predicted 298 TAAs were screened in BLAST for homology to HIV-1 peptide sequences. In total, 25 HIV-1 peptides were identified with sequence homology to predicted TAAs and showing high binding affinity to HLA alleles (i.e. < 100 nM) (Table 3). Interestingly, the highest number of HIV-1 peptides were identified for colon cancer (eleven) and breast cancer (six). Five peptides were identified for lung cancer, two for prostate cancer and only one for brain cancer (Fig. 2A). The broadest HLA coverage (6 alleles) was observed for the HIV peptides homologous to colon-specific TAAs, while the narrowest one (1 allele) was observed for the HIV peptides homologous to prostate-specific and brain-specific TAAs. Most importantly, only HIV peptides homologous to colon-, breast- and prostate-specific TAAs showed strong binding to HLA-A alleles (01:01; 02:01; 03:01 and 24:02) which are the most frequent in the world population (Fig. 2 B; Additional file 1: Fig. S3A; Additional file 1: Fig. S4). Considering the individual cancer-specific proteins, the highest number of HIV peptides homologous to TAAs (six) were predicted for the CCR9 (breast ca) and EPCAM (colon ca) proteins, covering the two most frequent HLA-A alleles in the world population (02:01 and 24:02) (Fig. 2 C; Additional file 1: Fig. S3B). The average binding affinity of such HIV peptides to the HLA alleles was very high (18.23 nM for A*02:01; 43,35 nM for A*24:02) (Fig. 2 D).

**Table 3:** HIV epitopes with homology to TuAs derived from cancer-specific proteins

Breast CA—CCR9						
HLA	TAA		HIV			
	PEPTIDE	AFF (nM)	PEPTIDE	AFF (nM)	Protein	GeneBank CODE
HLA-A*02:01	AIADLLFLV	3,76	RLRDLLFLV	10,01	env	AEN20914.1
HLA-B*58:01	LGCISQAQW	22,22	YCNISQAQW	30,01	env	QBF53490.1
			HCNISEAQW	66,16	env	AFE02648.1
			HCNISQTQW	61,58	env	QGJ15916.1
HLA-B*58:01	NSLVILVYW	28,08	ASLVIITYW	11,65	vif	AAW57764.1
HLA-A*24:02	CYTIIIHTL	40,22	NYTEIIHTL	43,35	env	AAX97247.1

Brain CA—DPYSL2						
HLA	TAA		HIV			
	PEPTIDE	AFF (nM)	PEPTIDE	AFF (nM)	Protein	GeneBank CODE
HLA-B*07:02	IPRRTTQRI	19,79	IPRRIRQRI	25,38	env	AET80771.1

Colon CA—CEACAM8						
HLA	TAA		HIV			
	PEPTIDE	AFF (nM)	PEPTIDE	AFF (nM)	Protein	GeneBank CODE
HLA-B*15:01	IQNPASANF*	9,34	RQGPASSNF	20,55	pol	QIC94847.1
HLA-B*58:01	YSWSVNGTF	20,84	SSWNVNGTW	5,6	env	ANO43474.1
HLA-A*03:01	GTFQQYTQK*	35,5	RTFQQYKKK	42	env	ABA08367.1
HLA-B*15:01	VTRNDTGSY	94,41	VTRNDTSTY	71,3	env	AAO20547.1

Colon CA—GLOD5						
HLA	TAA		HIV			
	PEPTIDE	AFF (nM)	PEPTIDE	AFF (nM)	Protein	GeneBank CODE
HLA-A*03:01	KMWGRTLEK	3,88	TQWGRTLEK	90,05	env	ACD34677.1

Colon CA—EPCAM						
HLA	TAA		HIV			
	PEPTIDE	AFF (nM)	PEPTIDE	AFF (nM)	Protein	GeneBank CODE
HLA-B*58:01	CSERVRTYW	23,26	VSERLKTYW	20,72	env	AAO20591.1
HLA-A*02:01	VVAGIVVLV	48,75	VVAGIIALV	24,48	vpu	AUO72667.1
			VIAGIVALV	17,96	vpu	ABI50903.1
			VVASIVALV	36,96	vpu	QGU21448.1
HLA-B*40:01	AEIKEMGEM	66,18	AELGEMGEM	87,18	vpu	AEW28038.1
HLA-B*39:01	THSPNSRAV	81,49	THSPSSREL	51,06	pol	AMX26992.1

Lung CA—PRPF40A						
HLA	TAA		HIV			
	PEPTIDE	AFF (nM)	PEPTIDE	AFF (nM)	Protein	GeneBank CODE
HLA-B*08:01	QLRKRNWEA	57,55	LLRRRGWEA	22,01	env	ACE65673.1
			VLRRRGWEA	43,08	env	AXP17656.1
			TLRRRGWEA	27,86	env	QMX89949.1
			ILRRRGWEA	35,96	env	QPK37756.1

Lung CA—CNIH4						
HLA	TAA		HIV			
	PEPTIDE	AFF (nM)	PEPTIDE	AFF (nM)	Protein	GeneBank CODE
HLA-B*58:01	LLNLPVATW	99,66	HCNLSVATW	38,73	env	ACR83664.1

Prostate CA—NDUFS2						
HLA	TAA		HIV			
	PEPTIDE	AFF (nM)	PEPTIDE	AFF (nM)	Protein	GeneBank CODE
HLA-A*01:01	VSDGSSRPY	72,62	SSDNSSRPY	60,07	env	ANC69009.1
			GSDSTSRPY	99,17	env	AFV81777.1

*peptide found at the HLA Ligand Atlas eluted from normal tissues.

### In vitro analysis of binding affinity and stability to HLA-A*02:01 molecule

The confirmation of predicted binding of peptides to HLA alleles could be experimentally confirmed only for the A*0201. The TAP-deficient T2 cell line was loaded with the individual peptides from paired TAA and HIV peptides. In particular, the VVAGIVVLV TAA derived from the colon ca-associated EPCAM protein was compared with the VVAGIIALV and VIAGIVALV epitopes derived from the HIV-1 vpu protein. The analysis confirmed that each peptide bound the HLA-A*02:01 molecule inducing a dose-dependent increase in the HLA surface expression on T2 cells over the background.

**Fig. 3:**
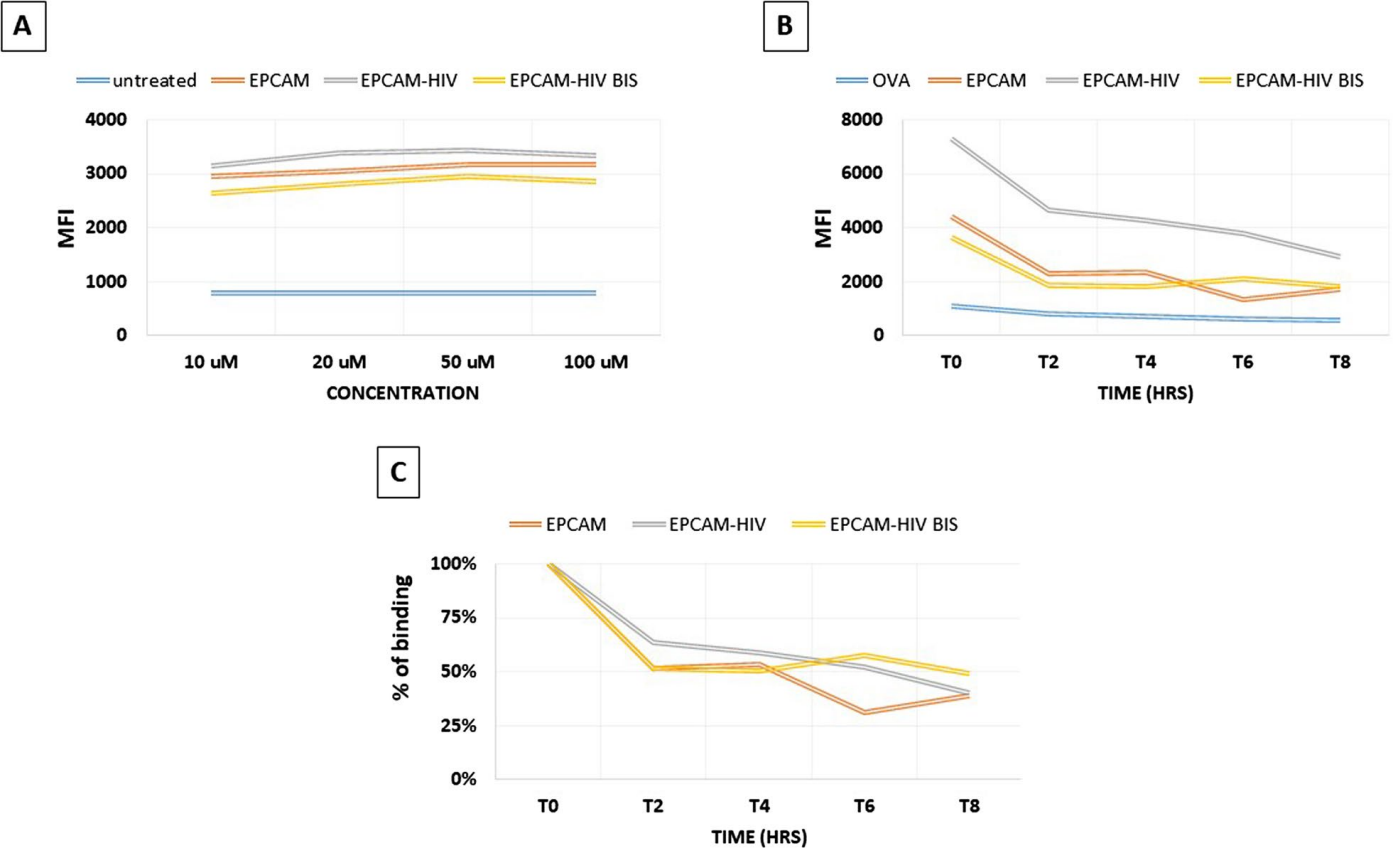
Experimental binding of TAAs and HIV-1 paired peptides to HLA-A*0201. Binding to HLA-A*0201 molecule and relative stability was assessed in TAP-deficient T2 cells loaded with the indicated peptides. Mean fluorescence intensity at flow cytometer indicates binding levels of peptides to HLA molecules (**A**). Decay of mean fluorescence intensity over time indicates stability of the peptide binding to the HLA molecule, expressed as absolute values (**B**) or percentage of values (**C**)

The results confirmed the comparable binding to HLA-A*02:01 of the three peptides as suggested by the predicted affinity values (Fig. 3A). Likewise, the peptide—MHC dissociation kinetics showed that the 50% dissociation value (Thalf) was reached 4 h after the peptide loading (T4), for the EPCAM TAA, and 6 – 8 h after the peptide loading (T8) for the two HIV peptides (Fig. 3B and C). Such results were representative of affinity and stability of the other HLA-A*02:01 associate peptides (data not shown).

### HIV peptide sequence conservation across viral isolates

HIV-1 is characterized by an extensive intra- and inter-host genome variability with generation of molecular “quasi-species” [23]. Therefore, the biological significance of the described homology between TAAs and HIV peptides is relevant only if the amino acid sequences are conserved across viral isolates. In order to assess such a sequence conservation, about 5000 HIV-1 sequences deposited in Blast were aligned to generate sequence logos. Indeed, logos provide the consensus sequence of all aligned peptides sequences as well as the relative frequency of amino acids at every position of the peptides. The results showed that the HIV peptides with the highest homology to TAAs identified in breast (CCR9), colon (EPCAM, GLOD5, CEACAM8) as well as prostate (NDUFS2) cancer and linked to most frequent HLA alleles (01:01; 02:01 and 24:02), are highly conserved across HIV isolates (Additional file 1: Fig. S5). In particular, HIV peptides RLRDLLFLV and NYTEIIHTL (homologous to breast CCR9) and VVAGIIALV (homologous to colon EPCAM) show 8 of 9 amino acids conserved in the consensus sequence. The HIV peptide SSDNSSRPY (homologous to prostate NDUFS2) shows identity in 7 of 9 amino acids compared to the consensus sequence.

Additional HIV peptides homologous to TAAs from breast and colon cancer and linked to less frequent HLA alleles show high conservation across HIV isolates. Finally, similar results are observed for the HIV peptide IPRRTTQRI (homologous to brain DPYSL2) showing identity in 7 of 9 amino acids compared to the consensus sequence. On the contrary, HIV peptides homologous to lung TAAs show poor identity (3 or 5 of 9 amino acids) to the consensus sequence (Additional file 1: Fig. S6).

### Peptide modelling and molecular docking

In order to verify that predicted paired TAAs and HIV-1 epitopes, including the HIV-1 consensus sequences, share contact residues with both the HLA molecule and the TCR α and β chains, epitope modelling and molecular docking were performed.

Epitopes crystallized with HLA alleles showing sequence homology with TAAs or HIV-1 peptides described in the present study were not found and examples of epitopes were used as templates to conduct the analyses. Crystallized pMHC complexes including also the TCR chains were available for the HLA-A*02:01, HLA-A*01:01, B*07:02, B*08:01.

**Fig. 4:**
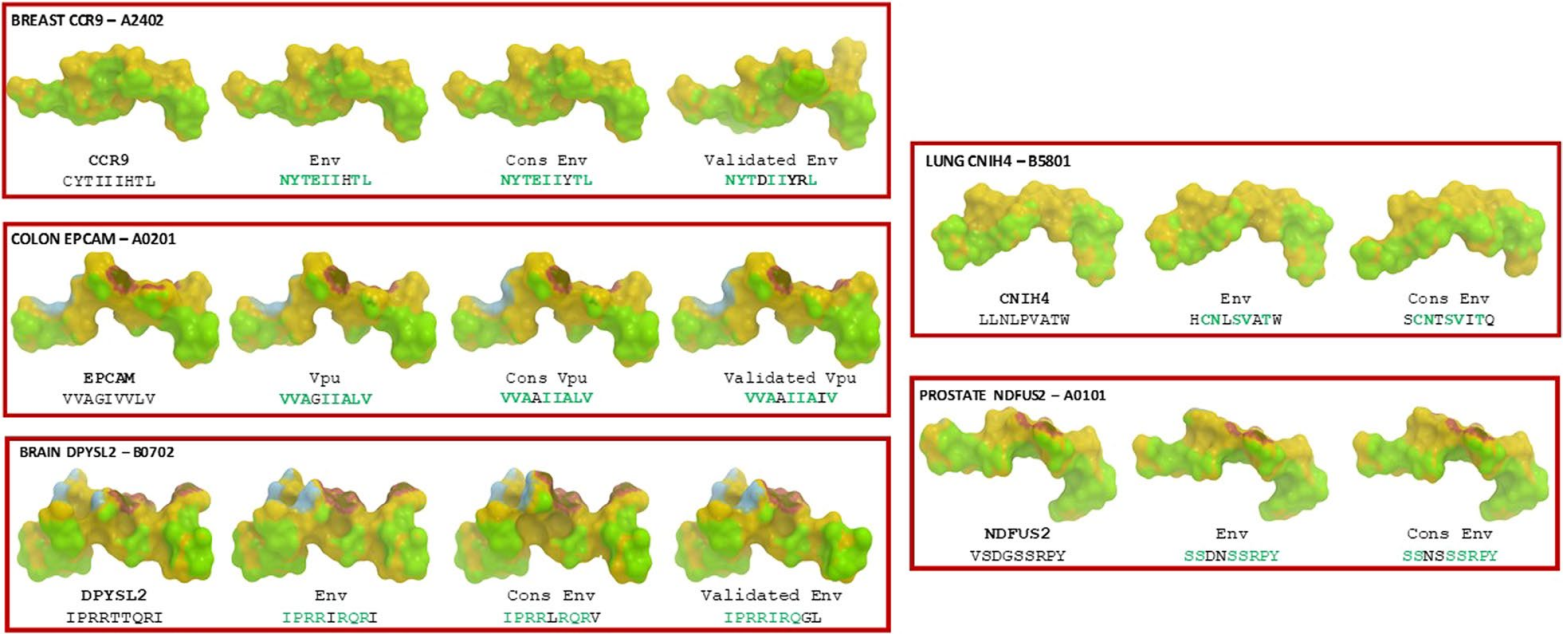
Predicted 3D conformation of paired peptides. The conformation of the TAAs and paired HIV-1 antigens and consensus bound to the indicated HLA molecules is shown. The prediction was performed using as template structure peptides crystallized with indicated HLA molecules. Only for HLA-A*02:01, HLA-A*01:01 and HLA-B*07:02 information about the β2 microglobulin, the α and β chains of the T cell receptor (TCR) were available (PDB https://www.rcsb.org/structure/1AO7, 5BRZ, 6VMX). Green areas = contact points with HLA molecule; Blue areas = contact points with the TCR α chain; Violet areas = contact points with the TCR β chain

As predictable from the sequence homology, the HIV peptides homologous to TAAs derived from breast, colon and prostate cancers showed highly similar, if not identical, conformation and contact points with HLA molecule and TCR chains as compared to corresponding TAAs. In detail, the breast CCR9 CYTIIIHTL and the HIV- 1 NYTEIIHTL show a conservative Cys to Asn and a non-conservative Ile to Glu substitutions in p1 and p4, respectively. The colon EPCAM VVAGIVVLV and the HIV- 1 VVAGIIALV show conservative Ile to Val and Val to Ala substitutions in p6 and p7, respectively. The prostate NDFUS2 VSDGSSRPY and the HIV-1 SSDNSSRPY show non-conservative Val to Ser and Gly to Asn substitutions in p1 and p4, respectively. They all do not induce significant conformational changes in the structure of paired epitopes. Moreover, the consensus sequences highly conserved across HIV isolates showed identical structure to individual HIV-1 peptides. In detail, the CCR9-like NYTEIIHTL peptide and the consensus NYTEIIYTL show a single non-conservative His to Tyr substitution in p7. The EPCAM-like VVAGIIALV peptide and the consensus VVAAIIALV show a single conservative Gly to Ala substitution in p4. The NDFUS2-like SSDNSSRPY peptide and the consensus SSNSSSRPY show a non-conservative Asp to Asn and a conservative Asn to Ser substitutions in p3 and p4, respectively (Fig. 4).

On the contrary, HIV peptides homologous to TAAs derived from brain and lung cancers did not show such high conformational similarities. In particular, the brain DPYSL2 IPRRTTQRI and the HIV-1 IPRRIRQRI show two non-conservative Thr to Ile and Thr to Arg substitutions in p5 and p6, respectively. The lung CNIH4 LLNLPVATW and the HIV-1 HCNLSVATW show three non-conservative Leu to His, Leu to Cys and Pro to Ser substitutions in p1, p2 and p5, respectively. They all induce significant conformational changes in the structure of paired epitopes. Moreover, the HIV consensus sequences showed structures significantly different from the individual HIV-1 peptides. In detail, the DPYSL2-like IPRRIRQRI peptide and the consensus IPRRLRQRV show two conservative Ile to Leu and Ile to Val substitutions in p5 and p9, respectively. The CNIH4-like HCNLSVATW peptide and the consensus SCNTSVITQ show three non- conservative His to Ser; Leu to Thr; Trp to Gln substitutions in p1, p4 and p9, respectively; and a conservative Ala to Ile substitution in p7 (Fig. 4). The Los Alamos HIV immunological database was interrogated for experimentally validated HIV epitopes showing sequence homology to those paired to TAAs. Indeed, validated epitopes with such characteristics were found for the breast ca CCR9 CYTIIIHTL epitope (NYTDIIYRL) [24], the brain ca DPYSL2 IPRRTTQRI epitope (IPRRIRQGL) [25] and the colon ca EPCAM VVAGIVVLV epitope (VVAAIIAIV) [26] (Fig. 4). In particular, the latter validated HIV epitope shows a relevant structural and conformational homology to the EPCAM TAA, confirming the biological relevance of the predictive findings. All other HIV peptides homologous to TAAs showed limited and variable similarity in conformation and contact points with HLA molecule and TCR chains as compared to corresponding TAAs (Additional file 1: Fig. S7–S9).

### *Identification of cross-reactive epitope-specific CD8* + *T cells*

In order to confirm the antigenicity of the predicted peptides and the cross-reactivity with the paired TAAs and HIV-1 peptides, we performed an analysis to detect reactive CD8^+^ specific T cells in PBMCs from HIV patients and healthy subjects (Additional file 1: Fig. S10).

An “ex vivo” expansion of HIV-specific T cells was induced culturing PBMCs with HIV peptides for 7 days. Subsequently, cells were incubated with tetramers loaded with the corresponding TAAs and analysed at cytofluorimeter.

Given the overwhelming prevalence of HLA-A*02:01 and 24:02 alleles in the cohort of enrolled HIV patients (16/22, 73%) (Additional file 2: Table S1), the analysis was performed with the paired HIV/TAA peptides from the EPCAM colon ca (HLA-A*02:01) and from the CCR9 breast ca (HLA-A*24:01) proteins.

**Fig. 5:**
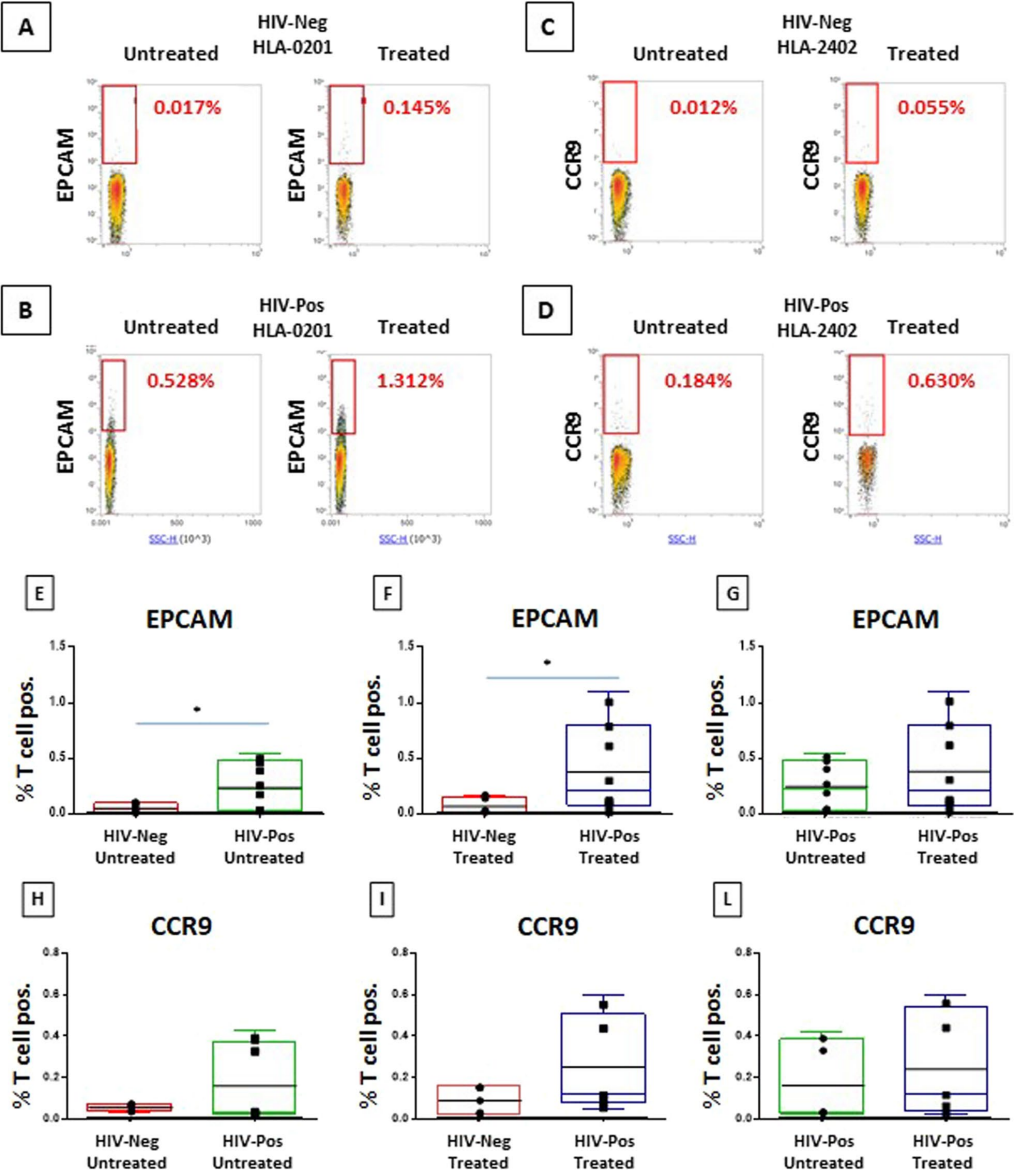
Evaluation of cross-reactive CD8 + T cells. pMHC directed expansion of T cells validated the presence of T cells specific for each paired TAA and viral epitopes are shown. Frequencies of pMHC multimer specific T cells out of total CD3^+^ CD8^+^ are displayed. Data from individual HIV patients and healthy donors are shown (**A**–**D**). Cumulative data from all HIV patients and all healthy donors are shown (**E**–**L**)

Strikingly, results show that HIV-positive subjects cross-react against HLA-A matched TAAs at baseline, even without any prior “ex vivo” expansion, while HIV-negative subjects do not (Fig. 5A and C). Such cross-reactivity is further increased only in HIV-positive subjects after the “ex vivo” expansion with the paired HIV peptide (Fig. 5B and D). The difference between the HIV-positive and -negative groups reaches the statistical significance for the EPCAM colon cancer HLA-A*02:01 paired peptides. The limited number of HLA-A*24:02 HIV-positive subjects does not provide the sufficient statistical power for the trend observed with CCR9 breast cancer paired peptides. The increased cross-reactivity against the TAA in HIV-positive subjects, prior and after the “ex vivo” expansion with HIV peptides, shows a clear trend that does not reach the statistical significance (Fig. 5E–L). No cross-reactivity was observed when T cells from HIV patients were reacted with HLA-matched not-homologous epitope (data not shown).

## Discussion

In the present study we aimed at identifying evidences for a molecular mimicry possibly explaining the strikingly reduced occurrence of breast, prostate and colon cancers in PLWHA reported by several meta-analyses. Indeed, a sequence and structural homology between HIV epitopes and tumor-antigens expressed by such cancers would provide an experimental proof of immunological cross-reactivity. Ultimately, the HIV infection would represent a natural “anti-cancer vaccination” eliciting a memory T cells response able to cross-react with tumor antigens and prevent/delay tumor growth.

The entire Normal Tissue and the Pathology datasets at the Human Protein Atlas (15,170 and 15,313 unique proteins, respectively) were sequentially interrogated to identify relevant cancer-specific proteins. In particular, such proteins should be highly expressed in cancer cells, not expressed in the normal cellular counterpart as well as not detected in > 70% of normal cells of any tissue. The latter characteristic is relevant, considering the aim of focusing on target antigens specifically expressed on cancer cells and not on normal cells. According to such parameters, six cancer-specific proteins in colon cancer (CEACAM8, CDH17, GLOD5, PPM1E, TRIM16, EPCAM), one in breast cancer (CCR9) and none in prostate cancer were identified. In parallel, no cancer-specific proteins matching such characteristics were identified in brain and lung cancers, selected as control tumors that do not show reduced occurrence in PLWHA.

The NetMHCpan software was used to predict strong binders (SBs) to the 12 HLA alleles that altogether cover more than 70% of the World population (http://www.allelefrequencies.net). Only SBs with a very high affinity to HLA molecules (< 100 nM) have been selected for the present analysis, according to our previous studies [11, 12, 16]. A total of 298 SBs were predicted from the cancer-specific proteins selected for each cancer, with a high variability among cancers and the highest number was predicted for the HLA-A*02:01 allele. The vast majority (93.7%) of such SBs have never been identified in the HLA ligandome of normal cells, suggesting they should be considered *bona fide* tumor-associated antigens (TAAs).

The homology search in BLAST returned 25 HIV-1 peptides with high binding affinity to HLA alleles, showing sequence homology to predicted TAAs. Ninenteen (76%) of these HIV-1 peptides were identified for colon [11], breast [6] and prostate [2] cancers. Only 5 were identified in the control lung [5] and brain [1] cancers. More importantly, only HIV peptides homologous to colon-, breast- and prostate-specific TAAs showed strong binding to HLA-A alleles (01:01; 02:01; 03:01 and 24:02) which are the most frequent in the world population. Experimental binding and stability assays in TAP-deficient T2 cells confirmed the predictive bioinformatics analysis. These results show that a high number of HIV-1 epitopes, homologous to TAAs, are found only for the three cancers with reduced occurrence in PLWHA. Moreover, they are associated with the most frequent HLA alleles, supporting the concept that their immunological protective role would cover most of the World population. In addition, the biological relevance of such observation is further supported by the sequence logos showing that TAAs-like HIV peptides in colon, breast and prostate cancers are highly conserved across HIV isolates. Indeed, this would confirm that the immune response elicited by HIV peptides would have an anti-cancer protective role regardless the high genetic variability of HIV-1 isolates.

Epitope modelling and molecular docking bioinformatics analyses confirmed that HIV peptides, and the corresponding consensus sequences, homologous to TAAs derived from breast, colon and prostate cancers showed highly similar, if not identical, conformation and contact points with HLA molecule and TCR chains as compared to corresponding TAAs. Such a similarity was not observed for the HIV peptides homologous to TAAs derived from lung and brain cancers.

The cross-reactivity to TAAs was ultimately demonstrated in ex vivo binding assays. Indeed, only PBMCs from HIV-positive patients, and not those from HIV-negative controls, reacted against HLA-matched TAAs. This confirmed that only HIV-positive patients have a circulating memory T cell population elicited by HIV peptides homologous to cancer-specific TAAs. The identification of validated HIV epitopes with a relevant structural and conformational homology to predicted epitopes, strongly confirms the biological relevance of our findings.

Overall, the present study shows for the first time the molecular mimicry between HIV epitopes and TAAs derived from cancer-specific proteins associated with colon, breast and prostate cancers that show reduced occurrence in PLWHA. All the experimental evidences strongly suggest that this could be, in fact, the immunological explanation for such epidemiological observation. Therefore, HIV infection may represent a “natural anti-cancer vaccination” eliciting a memory T cell compartment able to cross-react with cancer cells and providing a protection from cancer development and progression. This represents a breakthrough in cancer immunology with highly relevant implications beyond HIV infection.

Two independent studies have recently reported evidence for cancer regression in SARS-CoV2 infected or vaccinated individuals. No experimental evidence for SARS-CoV2 epitopes sharing sequence or structural homology with TAAs has been shown. Nevertheless, such clinical evidences support for a broader interplay between viral and cancer T cell responses [27, 28].

Other more ubiquitous viral infections may play a role as “natural anti-cancer vaccination” and a much larger population may be protected from development and progression of different cancers. In addition, viral antigens homologous to TAAs may be used in anti-cancer preventive/therapeutic vaccine formulations with higher antigenicity and immunogenicity than over-expressed tumor self-antigens.

The present study ultimately provides a definitive explanation of the reduced occurrence of breast, prostate and colon cancers in PLWHA and represents a new way of thinking in cancer immunology.

## Contribution to the field statement

### Evidence before this study

We and others have previously shown that the molecular mimicry between viral and tumor antigens may have a significant impact in controlling tumor growth and improving the clinical outcome in cancer patients. More recently, we have shown that TAAs may show sequence homology as well as structural similarities with viral peptides and cross-reacting CD8^+^ T cells can be identified to drive the fate of cancer development and progression. In particular, such homologies must be found at the residues facing the T cell receptor (TCR). Therefore, a previous viral infection may represent a natural “anti-cancer vaccination”.

### Added value of this study

This is the first study showing a molecular mimicry between HIV antigens and TAAs identified in breast, prostate and colon cancers. T cells cross-reacting with paired antigens has been demonstrated.

### Implications of all the available evidence

Therefore, it is highly reasonable that memory CD8^+^ T cells elicited during the HIV infection may play a key role in controlling development and progression of such cancers in the PLWHA lifetime. This represents the first demonstration ever that a viral infection may induce natural “preventive” anti-cancer memory T cells, with highly relevant implications beyond the HIV infection.

## Supplementary Information

**Additional file 1: Figure S1**. Cancer specific proteins. Indicated cancer-specific proteins are highly expressed in each cancer and not expressed in normal cells of the same tissue. The percentage of not detection in all normal cells is shown. **Figure S2**. Predicted tumor antigens. **Figure S3.** Number of HIV-1 predicted antigens associated to each HLA allele homologous to TAAs listed by HLA allele (A). Number of HIV-1 predicted antigens associated to each HLA allele homologous to TAAs from each cancer-specific proteins identified in each tumor, listed HLA allele (B). **Figure S4.** Worldwide alleles frequencies. **Figure S5. **Sequence Logos of HIV epitopes. **Figure S6.** Sequence Logos of HIV epitopes. **Figure S7.** Predicted 3D conformation of paired peptides for BREAST ca. **Figure S8.** Predicted 3D conformation of paired peptides for COLON ca. **Figure S9**. Predicted 3D conformation of paired peptides for BRAIN and LUNG ca. **Figure S10.** Gating strategy for selection of CD8+ T cells.

**Additional file 2: Table S1.** HLA typing of HIV-1 patients enrolled in the study

## Data Availability

All data generated or analysed during this study are included in this published article and its supplementary information files. Raw data are available at the public repository: 10.5281/zenodo.7124230. The preprint is available at https://papers.ssrn.com/sol3/papers.cfm?abstract_id=4115467.

## Abbreviations

HIV: Human immunodeficiency virus
AIDS: Acquired Immunodeficiency Syndrome
PLWHA: People living with HIV /AIDS
TAA: Tumor associated antigen
TCR: T cell receptor
TME: Tumor microenvironment
TAA: Tumor-associated antigen
VirA: Viral antigen
MHC: Major histocompatibility complex
SIR: Standardized incidence ratio
DMSO: Dimethyl sulfoxide
PBS: Phosphate Buffered Saline
TAP: Transporter associated with antigen processing
OVA: Ovalbumin
FI: Fluorescence index
MFI: Mean fluorescence intensity
pMHC: PeptideMHC
PBMCs: Peripheral blood mononuclear cells
SB: Strong binder

## References

[1] Ross AL, Bråve A, Scarlatti G, Manrique A, Buonaguro L (2010). Progress towards development of an HIV vaccine: report of the AIDS Vaccine 2009 Conference. Lancet Infect Dis.

[2] Biggar RJ, Chaturvedi AK, Goedert JJ, Engels EA (2007). AIDS-related cancer and severity of immunosuppression in persons with AIDS. J Natl Cancer Inst.

[3] Chiao EY, Coghill A, Kizub D, Fink V, Ndlovu N, Mazul A (2021). The effect of non-AIDS-defining cancers on people living with HIV. Lancet Oncol.

[4] Grulich AE, van Leeuwen MT, Falster MO, Vajdic CM (2007). Incidence of cancers in people with HIV/AIDS compared with immunosuppressed transplant recipients: a meta-analysis. Lancet.

[5] Shiels MS, Cole SR, Kirk GD, Poole C (2009). A meta-analysis of the incidence of non-AIDS cancers in HIV-infected individuals. J Acquir Immune Defic Syndr.

[6] Grulich AE, Vajdic CM (2015). The epidemiology of cancers in human immunodeficiency virus infection and after organ transplantation. Semin Oncol.

[7] Coghill AE, Engels EA, Schymura MJ, Mahale P, Shielset MS (2018). Risk of breast, prostate, and colorectal cancer diagnoses among HIV-infected individuals in the United States. J Natl Cancer Inst.

[8] Buonaguro L, Cerullo V (2021). Pathogens: our allies against cancer?. Mol Ther.

[9] Snyder A, Makarov V, Merghoub T, Yuan J, Zaretsky JM, Desrichard A (2014). Genetic basis for clinical response to CTLA-4 blockade in melanoma. N Engl J Med.

[10] Balachandran VP, Łuksza M, Zhao JN, Makarov V, Moral JA, Remark R (2017). Identification of unique neoantigen qualities in long-term survivors of pancreatic cancer. Nature.

[11] Petrizzo A, Tagliamonte M, Mauriello A, Costa V, Aprile M, Esposito R (2018). Unique true predicted neoantigens (TPNAs) correlates with anti-tumor immune control in HCC patients. J Transl Med.

[12] Ragone C, Manolio C, Cavalluzzo B, Mauriello A, Tornesello ML, Buonaguro FM (2021). Identification and validation of viral antigens sharing sequence and structural homology with tumor-associated antigens (TAAs). J Immunother Cancer.

[13] Wooldridge L, Ekeruche-Makinde J, van den Berg HA, Skowera A, Miles JJ, Tan MP (2012). A single autoimmune T cell receptor recognizes more than a million different peptides. J Biol Chem.

[14] Sewell AK (2012). Why must T cells be cross-reactive?. Nat Rev Immunol.

[15] Reynisson B, Alvarez B, Paul S, Peters B, Nielsen M (2020). NetMHCpan-4.1 and NetMHCIIpan-4.0: improved predictions of MHC antigen presentation by concurrent motif deconvolution and integration of MS MHC eluted ligand data. Nucleic Acids Res.

[16] Cavalluzzo B, Mauriello A, Ragone C, Manolio C, Tornesello ML, Buonaguro FM (2021). Novel molecular targets for hepatocellular carcinoma. Cancers (Basel).

[17] Saini SK, Tamhane T, Anjanappa R, Saikia A, Ramskov S, Donia M (2019). Empty peptide-receptive MHC class I molecules for efficient detection of antigen-specific T cells. Sci Immunol.

[18] Neidert MC, Kowalewski DJ, Silginer M, Kapolou K, Backert L, Freudenmann LK (2018). The natural HLA ligandome of glioblastoma stem-like cells: antigen discovery for T cell-based immunotherapy. Acta Neuropathol.

[19] Liu K-J, Wang C-C, Chen L-T, Cheng A-L, Lin D-T, Wu Y-C (2004). Generation of carcinoembryonic antigen (CEA)-specific T-cell responses in HLA-A*0201 and HLA-A*2402 late-stage colorectal cancer patients after vaccination with dendritic cells loaded with CEA peptides. Clin Cancer Res.

[20] Ruiz M, Kobayashi H, Lasarte JJ, Prieto J, Borrás-Cuesta F, Celis E (2004). Identification and characterization of a T-helper peptide from carcinoembryonic antigen. Clin Cancer Res.

[21] Choi YJ, Park S-J, Park Y-S, Park HS, Yang KM, Heo K (2018). EpCAM peptide-primed dendritic cell vaccination confers significant anti-tumor immunity in hepatocellular carcinoma cells. PLoS ONE.

[22] Bassani-Sternberg M, Chong C, Guillaume P, Solleder M, Pak H, Gannon PO (2017). Deciphering HLA-I motifs across HLA peptidomes improves neo-antigen predictions and identifies allostery regulating HLA specificity. PLoS Comput Biol.

[23] Fisher W, Giorgi EE, Chakraborty S, Nguyen K, Bhattacharya T, Theiler J (2021). HIV-1 and SARS-CoV-2: Patterns in the evolution of two pandemic pathogens. Cell Host Microbe.

[24] Barton JP, Goonetilleke N, Butler TC, Walker BD, McMichael AJ, Chakraborty AK (2016). Relative rate and location of intra-host HIV evolution to evade cellular immunity are predictable. Nat Commun.

[25] Brockman MA, Kwon DS, Tighe DP, Pavlik DF, Rosato PC, Sela J (2009). IL-10 is up-regulated in multiple cell types during viremic HIV infection and reversibly inhibits virus-specific T cells. Blood.

[26] Corbet S, Nielsen HV, Vinner L, Lauemoller S, Therrien D, Tang S (2003). Optimization and immune recognition of multiple novel conserved HLA-A2, human immunodeficiency virus type 1-specific CTL epitopes. J Gen Virol.

[27] Sousa LGD, McGrail DJ, Li K, Marques-Piubelli ML, Gonzalez C, Dai H (2022). Spontaneous tumor regression following COVID-19 vaccination. J Immuno Therap Cancer.

[28] Ottaiano A, Scala S, D'Alterio C, Trotta A, Bello A, Rea G (2021). Unexpected tumor reduction in metastatic colorectal cancer patients during SARS-Cov-2 infection. Ther Adv Med Oncol.

