# Supplementary material for: Antigenic molecular mimicry in viral-mediated protection from cancer: the HIV case
Source: J Transl Med. 2022 Oct 15;20:472. doi: 10.1186/s12967-022-03681-4 (PMC9569184; doi:10.1186/s12967-022-03681-4)
Supplement: Supplementary file 1 — Additional file 1: Figure S1. Cancer specific proteins. Indicated cancer-specific proteins are highly expressed in each cancer and not expressed in normal cells of the same tissue. The percentage of not detection in all normal cells is shown. Figure S2. Predicted tumor antigens. Figure S3. Number of HIV-1 predicted antigens associated to each HLA allele homologous to TAAs listed by HLA allele (A). Number of HIV-1 predicted antigens associated to each HLA allele homologous to TAAs from each cancer-specific proteins identified in each tumor, listed HLA allele (B). Figure S4. Worldwide alleles frequencies. Figure S5. Sequence Logos of HIV epitopes. Figure S6. Sequence Logos of HIV epitopes. Figure S7. Predicted 3D conformation of paired peptides for BREAST ca. Figure S8. Predicted 3D conformation of paired peptides for COLON ca. Figure S9. Predicted 3D conformation of paired peptides for BRAIN and LUNG ca. Figure S10. Gating strategy for selection of CD8+ T cells. [file 12967_2022_3681_MOESM1_ESM.pdf]

Suppl. Fig. 1

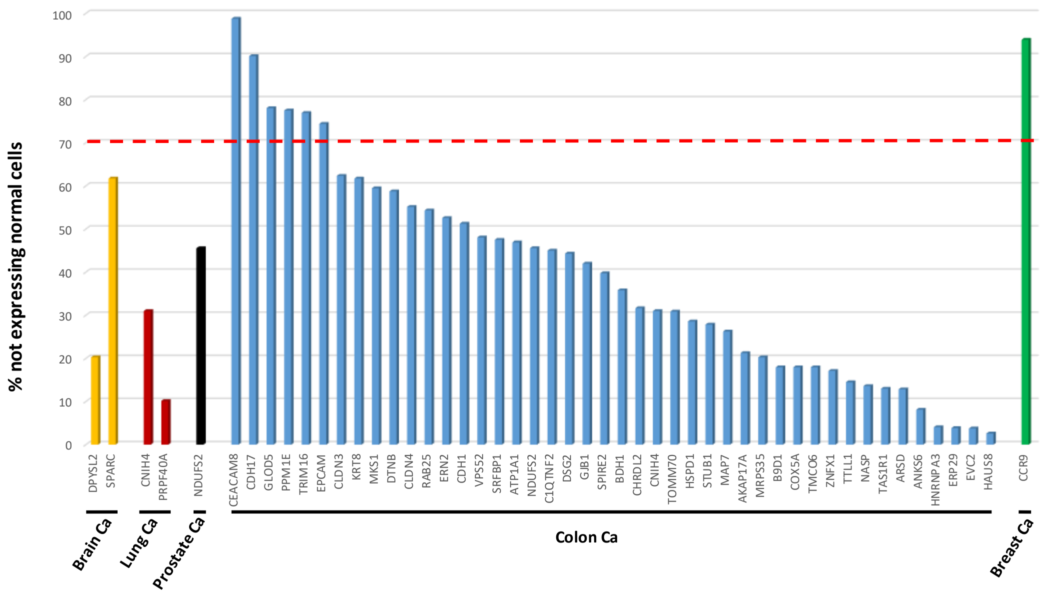

Suppl. Fig. 1 Cancer specific proteins. Indicated cancer-specific proteins are highly expressed in each cancer and not expressed in normal cells of the same tissue. The percentage of not detection in all normal cells is shown.

Suppl. Fig. 2

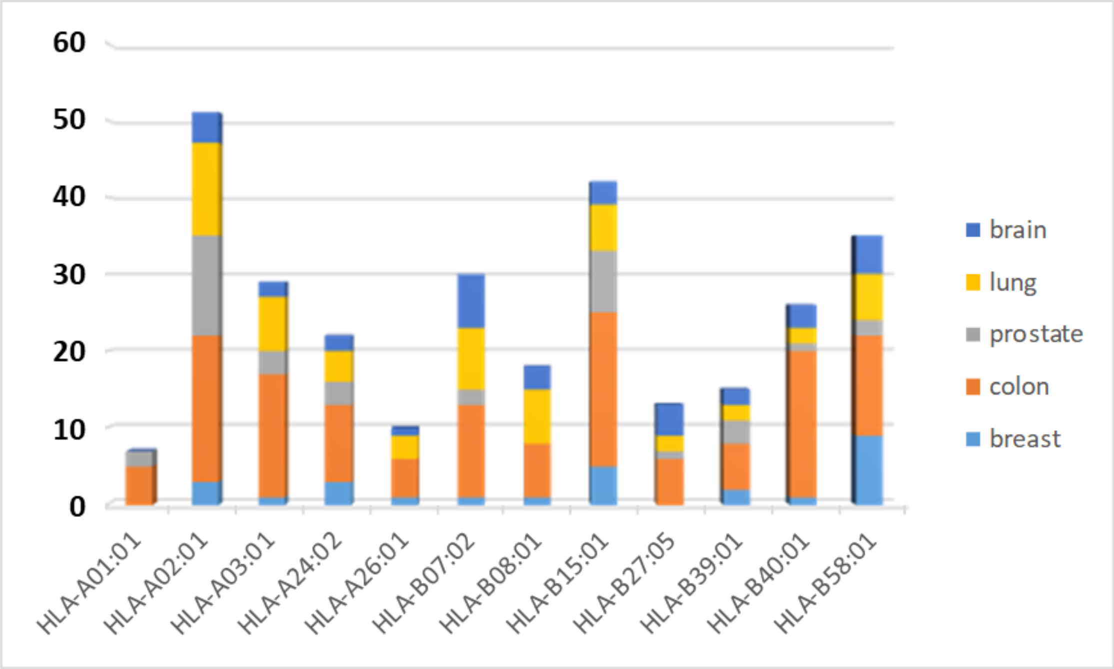

Suppl. Fig. 2 Predicted tumor antigens. Cumulative number of predicted antigens from all cancer-specific proteins identified in each tumor, listed by HLA-A allele.

Suppl. Fig. 3

A

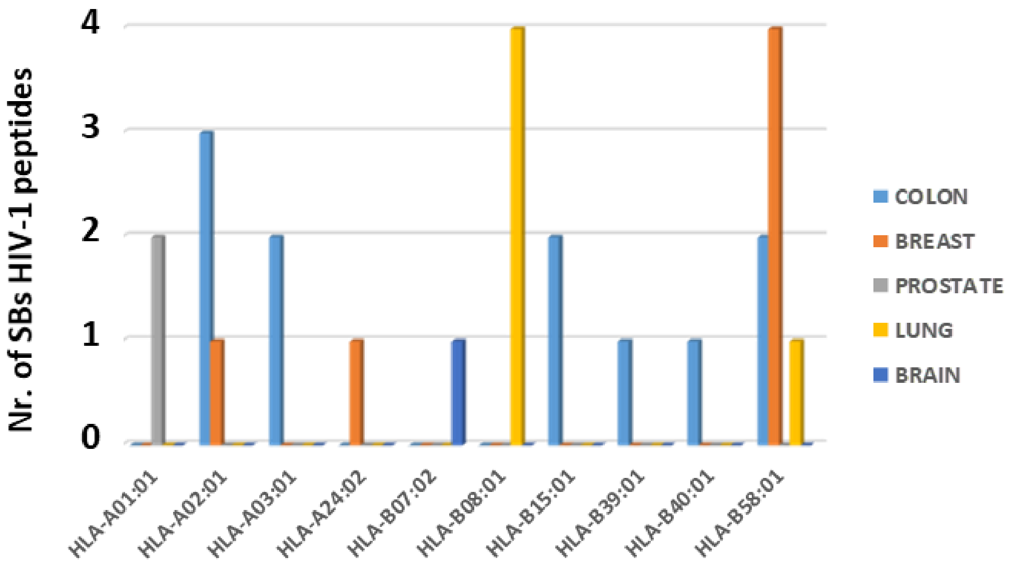

B

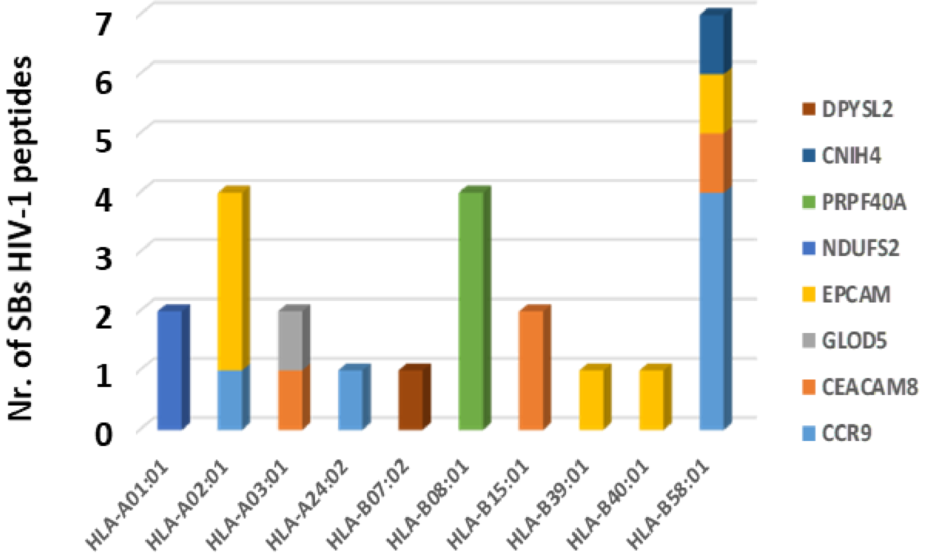

Suppl. Fig. 3 **Number of HIV-1 predicted antigens** associated to each HLA allele homologous to TAAs listed by HLA allele (A). Number of HIV-1 predicted antigens associated to each HLA allele homologous to TAAs from each cancer-specific proteins identified in each tumor, listed HLA allele (B)

Suppl. Fig. 4

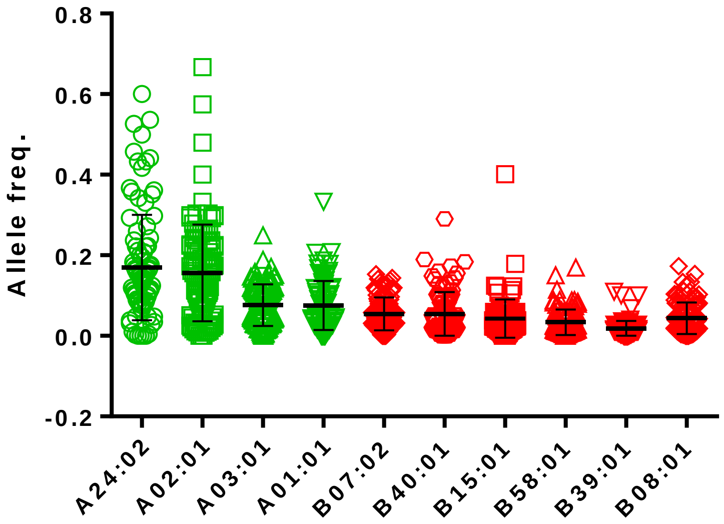

Suppl. Fig. 4 Worldwide alleles frequencies. The frequency of each allele analyzed in the study is reported. Each symbol is the frequency reported in a single cohort group (http://www.allelefrequencies.net/)

Suppl. Fig. 5

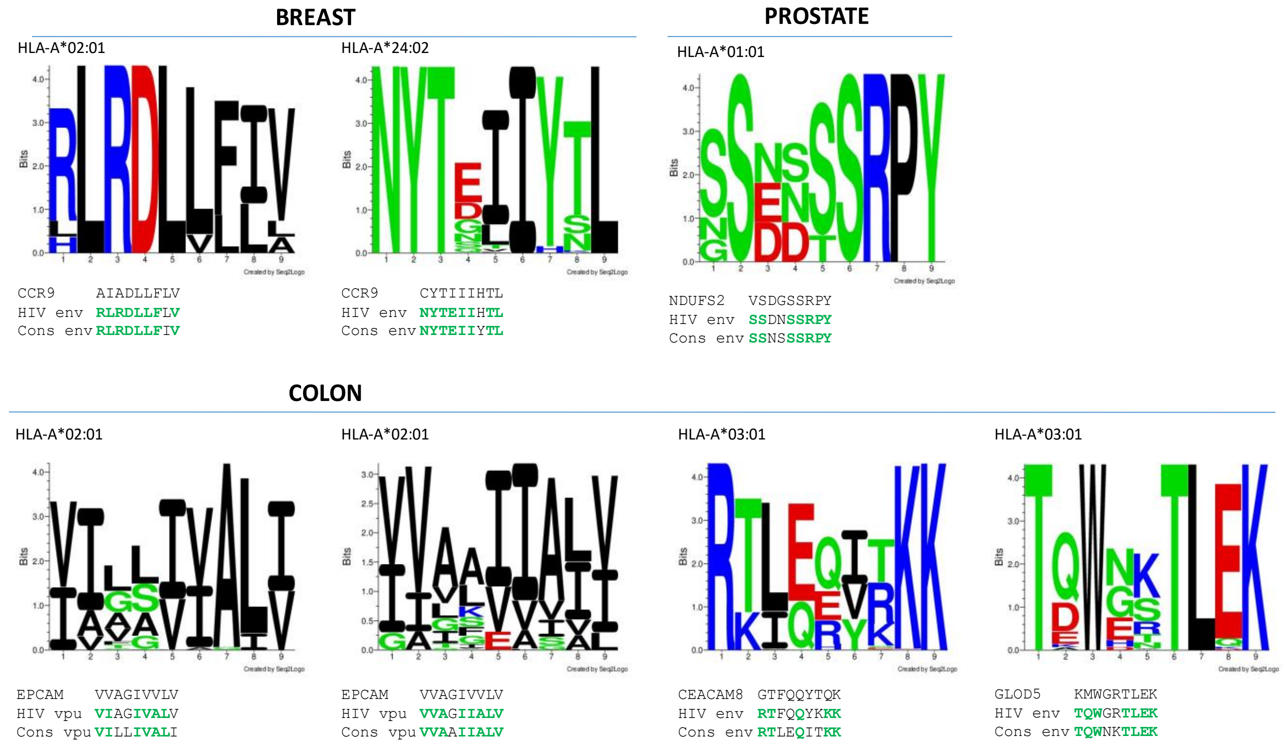

Suppl. Fig. 5 Sequence Logos of HIV epitopes. Amino acid sequences from 5000 HIV isolates were selected and piled up to build sequence logos. The height of the stack indicates the sequence conservation at that position, while the height of symbols within the stack indicates the relative frequency of each amino or nucleic acid at that position (https://services.healthtech.dtu.dk/service.php?Seq2Logo-2.0).

Suppl. Fig. 6

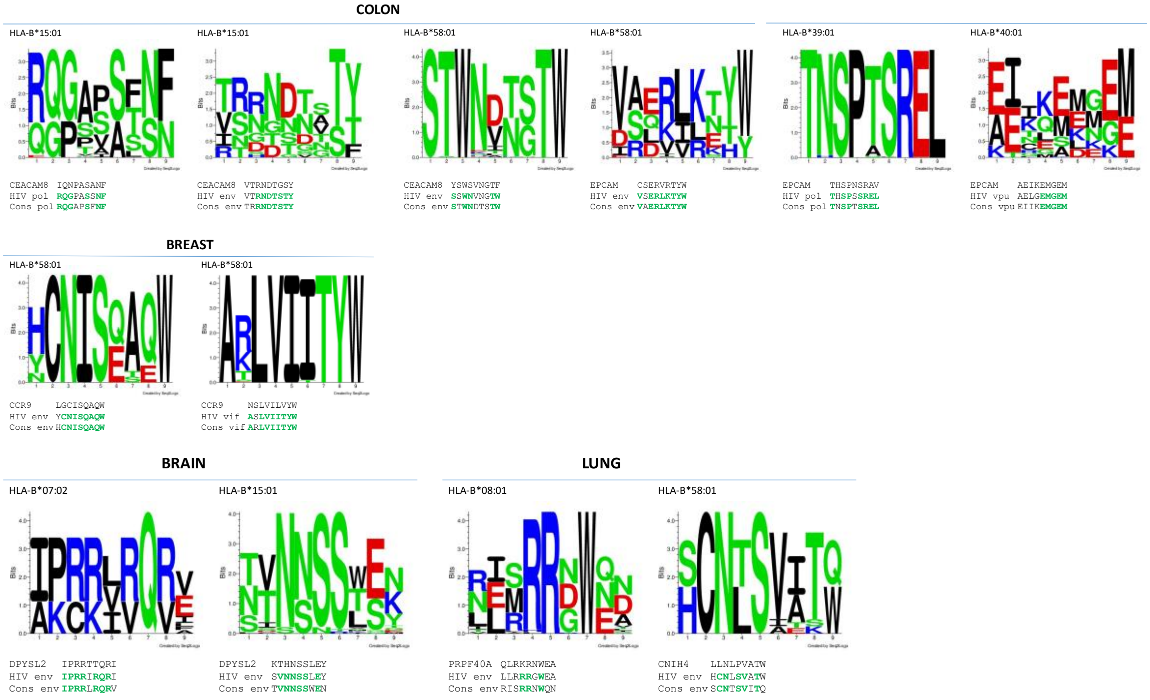

Suppl. Fig. 6 Sequence Logos of HIV epitopes. Amino acid sequences from 5000 HIV isolates were selected and piled up to build sequence logos. The height of the stack indicates the sequence conservation at that position, while the height of symbols within the stack indicates the relative frequency of each amino or nucleic acid at that position (https://services.healthtech.dtu.dk/service.php?Seq2Logo-2.0).

Suppl. Fig. 7

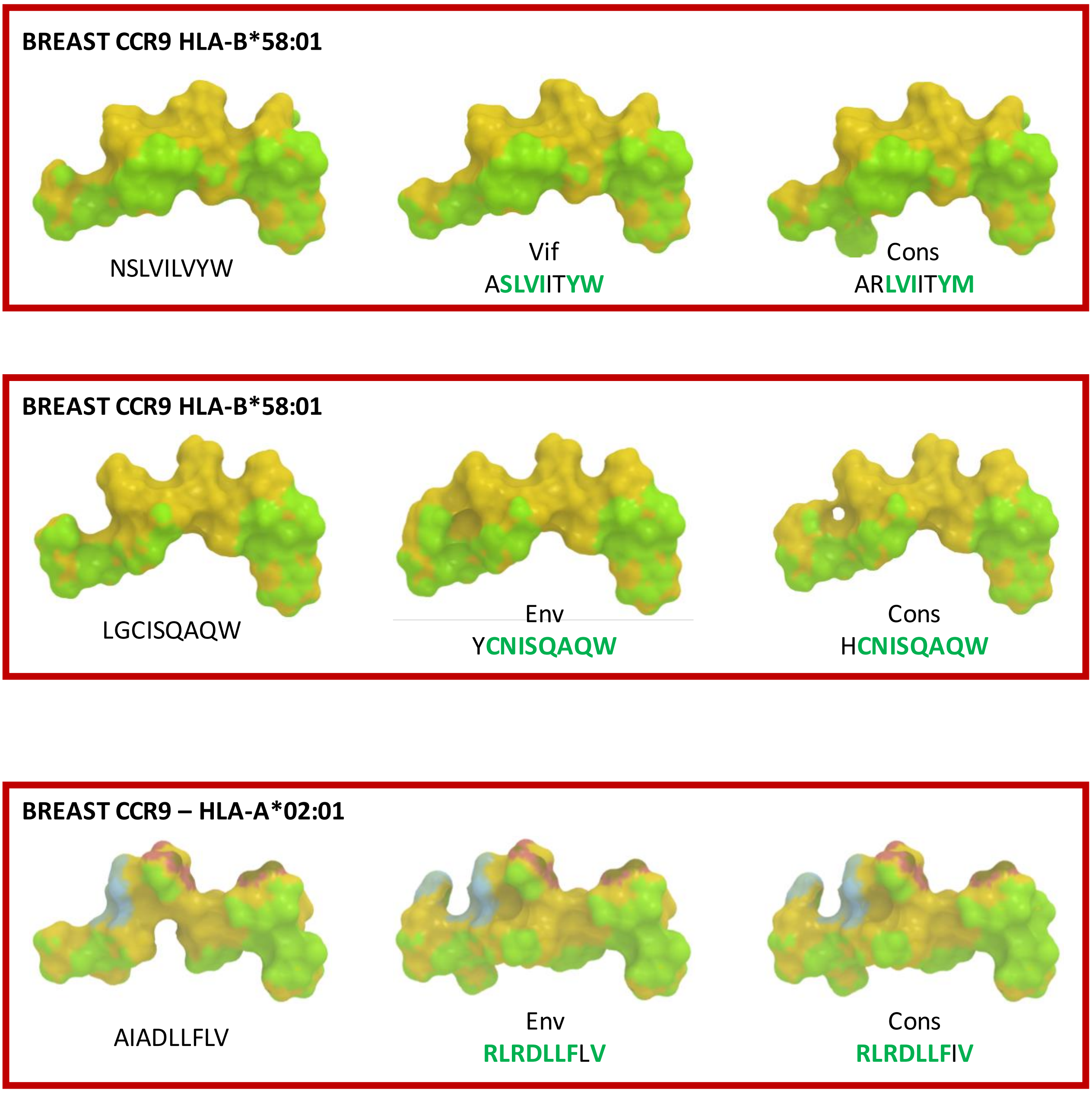

Suppl. Fig. 7 **Predicted 3D conformation of paired peptides for BREAST ca.** The conformation of the CCR9 TuAs and paired HIV-1 antigens and consensus bound to the indicated HLA molecules is shown. The prediction was performed using as template structure peptides crystallized with indicated HLA molecules. Only for HLA-A*02:01 was available information about the β2 microglobulin, the α and β chains of the T cell receptor (TCR) (PDB https://www.rcsb.org/structure/1AO7). Green areas = contact points with HLA molecule; Blue areas=contact points with the TCR α chain; Violet areas=contact points with the TCR β chain

Suppl. Fig. 8

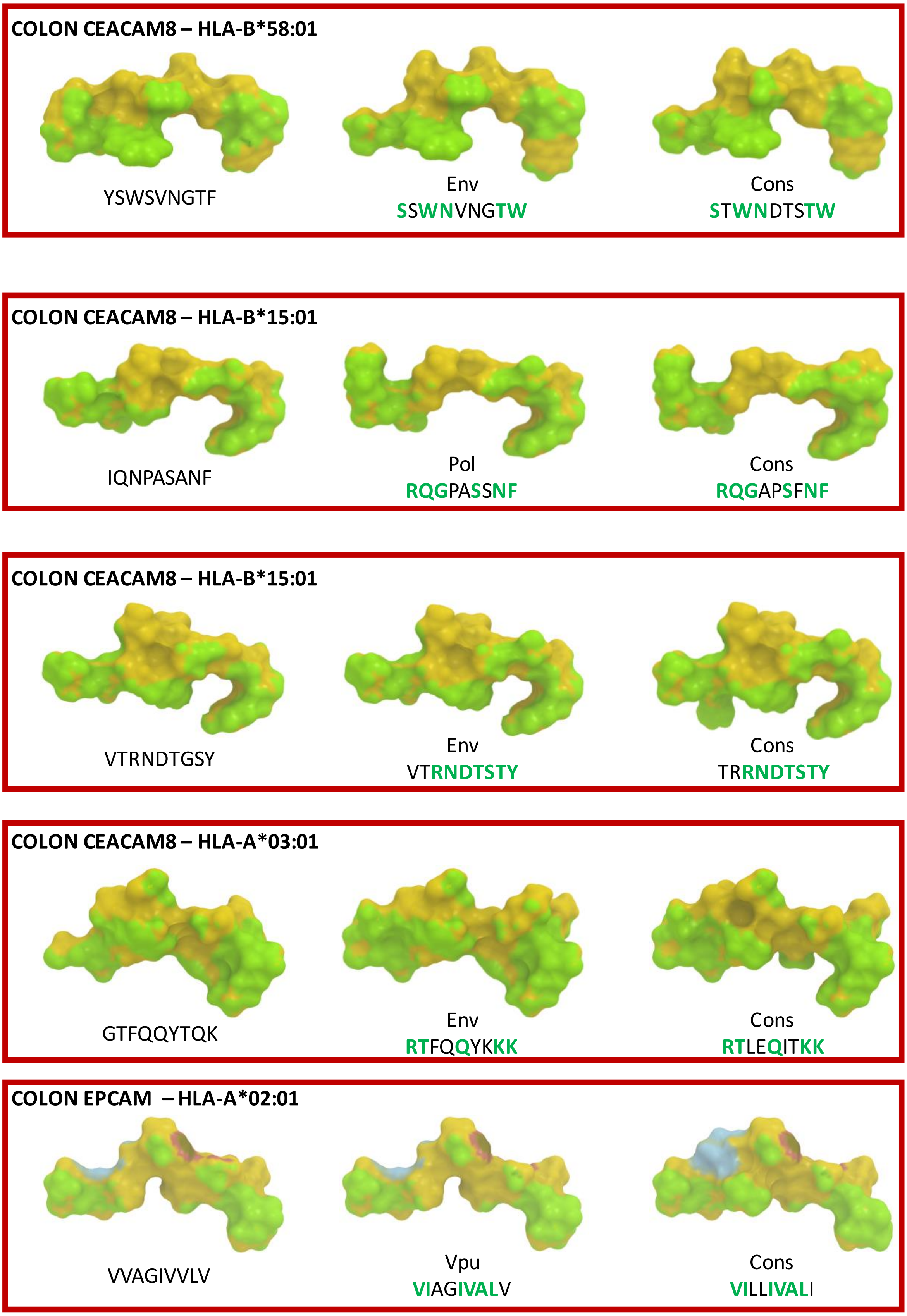
COLON CEACAM8 – HLA-B*58:01
YSWSVNGTF
Env
SSWNVNGTW
Cons
STWNDTSTW
COLON CEACAM8 – HLA-B*15:01
IQNPASANF
Pol
RQGPASSNF
Cons
RQGAPSFNF
COLON CEACAM8 – HLA-B*15:01
VTRNDTGSY
Env
VTRNDTSTY
Cons
TRRNDTSTY
COLON CEACAM8 – HLA-A*03:01
GTFQQYTQK
Env
RTFQQYKKK
Cons
RTLEQITKK
COLON EPCAM – HLA-A*02:01
VVAGIVVLV
Vpu
VIAGIVALV
Cons
VILLIVALI

Suppl. Fig. 8 cont.d

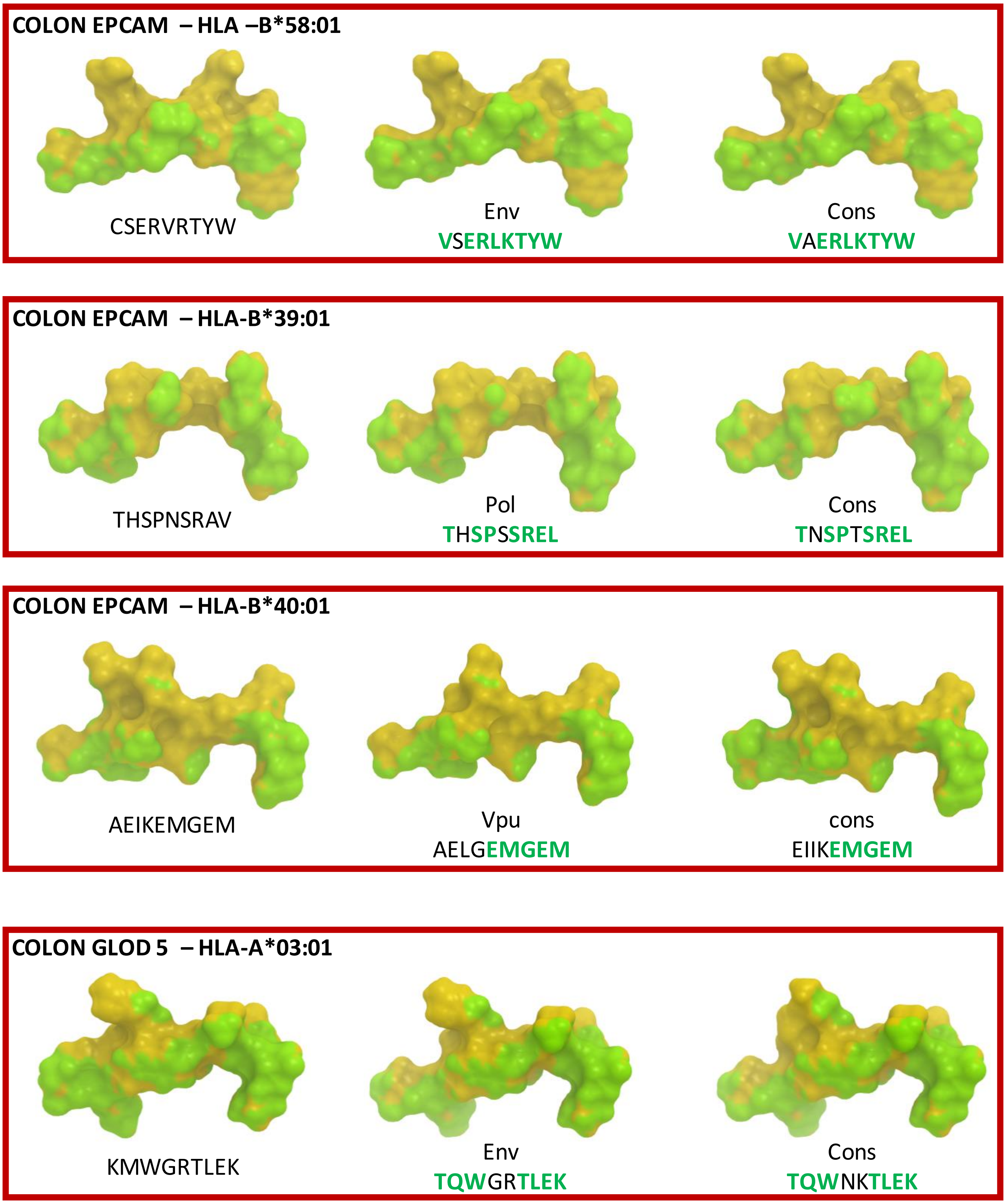

Suppl. Fig. 8 Predicted 3D conformation of paired peptides for COLON ca. The conformation of the CEACAM8 and EPCAM TuAs and paired HIV-1 antigens and consensus bound to the indicated HLA molecules is shown. The prediction was performed using as template structure peptides crystallized with indicated HLA molecules. Only for HLA-A*02:01 was available information about the β2 microglobulin, the α and β chains of the T cell receptor (TCR) (PDB https://www.rcsb.org/structure/1AO7). Green areas = contact points with HLA molecule; Blue areas=contact points with the TCR α chain; Violet areas=contact points with the TCR β chain

Suppl. Fig. 9

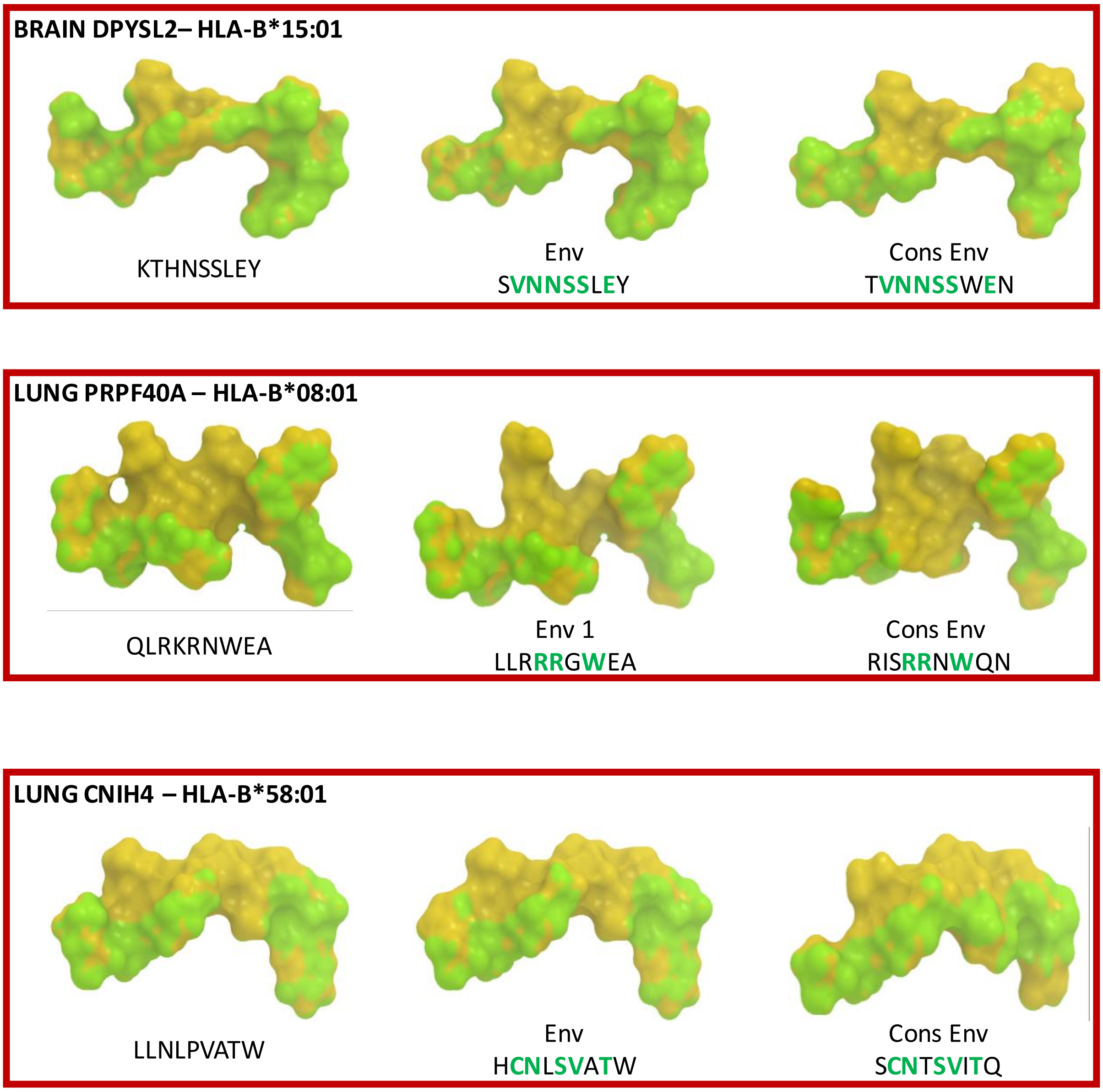

Suppl. Fig. 9 **Predicted 3D conformation of paired peptides for BRAIN and LUNG ca.** The conformation of the DPYSL2, PRPF40A and CNIH4 TuAs and paired HIV-1 antigens and consensus bound to the indicated HLA molecules is shown. The prediction was performed using as template structure peptides crystallized with indicated HLA molecules. Green areas = contact points with HLA molecule.

Suppl. Fig. 10

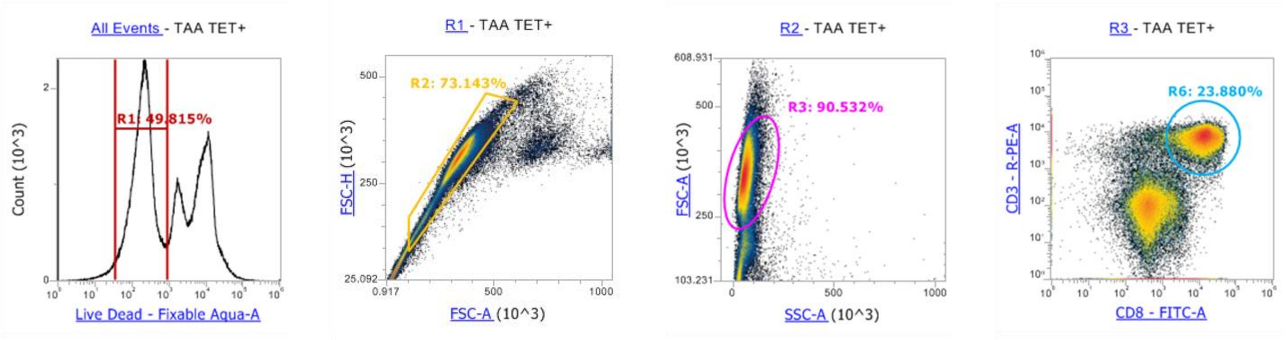

Suppl. Fig. 10 Gating strategy for selection of CD8⁺ T cells. Viable cells were gated based on negative expression of the fixable viability stain (FVS) (R1); b) selection was made to exclude doublets (R2); c) lymphocytes were gated based on forward (FSC) and side scatter (SSC) profiles (R3); d) gating for CD8 + T cells was performed according to CD3 and CD8 double positivity.
