# Supplementary material for: Antigenic molecular mimicry in viral-mediated protection from cancer: the HIV case
Source: J Transl Med. 2022 Oct 15;20:472. doi: 10.1186/s12967-022-03681-4 (PMC9569184; doi:10.1186/s12967-022-03681-4)
Supplement: Supplementary file 2 — Additional file 2: Table S1. HLA typing of HIV-1 patients enrolled in the study [file 12967_2022_3681_MOESM2_ESM.docx]

Suppl. Table 1. HLA typing of HIV-1 patients enrolled in the study.

| Sample ID | HLA-A | | HLA-B | | HLA-C | |
| --- | --- | --- | --- | --- | --- | --- |
| HIV-001 | 03:01 | 24:02 | 08:01 | 35:03 | 04:01 | 07:01 |
| HIV-002 | 01:01 | _ | 41:01 | 50:01 | 07:04 | 17:01 |
| HIV-003 | 03:01 | 29:01 | 08:01 | 41:02 | 07:01 | 17:03 |
| HIV-004 | 24:02 | 33:03 | 07:02 | 58:01 | 03:02 | 07:02 |
| HIV-005 | 02:01 | 24:02 | 18:01 | _ | 07:01 | 12:03 |
| HIV-006 | 01:01 | 02:01 | 35:03 | 51:01 | 04:01 | 16:XX |
| HIV-007 | 01:01 | 02:01 | 35:03 | 08:01 | 04:01 | 07:01 |
| HIV-008 | 02:05 | 24:02 | 41:01 | 44:06 | 05:01 | 17:01 |
| HIV-009 | 01:01 | 02:01 | 35:01 | _ | 04:01 | 06:02 |
| HIV-010 | 02:01 | 26:01 | 27:02 | 38:01 | 02:02 | 12:03 |
| HIV-011 | 26:01 | 33:01 | 14:02 | 35:01 | 08:02 | 16:02 |
| HIV-012 | _ | _ | _ | _ | _ | _ |
| HIV-013 | 32:01 | 68:02 | 44:02 | 47:03 | 05:01 | 07:18 |
| HIV-014 | 02:01 | 30:02 | 49:01 | 51:29 | 02:02 | 07:01 |
| HIV-015 | 01:01 | 24:02 | 18:01 | 35:03 | 05:01 | 12:03 |
| HIV-016 | 01:01 | 03:01 | 07:02 | 08:01 | 07:XX | _ |
| HIV-017 | _ | _ | _ | _ | _ | _ |
| HIV-018 | 24:02 | _ | 27:05 | _ | 01:XX | _ |
| HIV-019 | 02:01 | 24:03P | 18:01 | 49:01 | 07:01 | _ |
| HIV-020 | 29:01 | 30:01 | 35:03 | 39:01 | 04:01 | 12:03 |
| HIV-021 | 02:05 | 24:02 | 35:02 | 50:01 | 04:01 | 06:02 |
| HIV-022 | 01:01 | 02:01 | 51:01 | 55:01 | 02:02 | 03:03 |

HLA typing was not achieved in HIV-012 and 017 samples for poor DNA quality.
